# Supplementary figures and images for: Can Acropora tenuis larvae attract native Symbiodiniaceae cells by green fluorescence at the initial establishment of symbiosis?
Source: PLoS One. 2021 Jun 1;16(6):e0252514. doi: 10.1371/journal.pone.0252514 (PMC8168901; doi:10.1371/journal.pone.0252514)

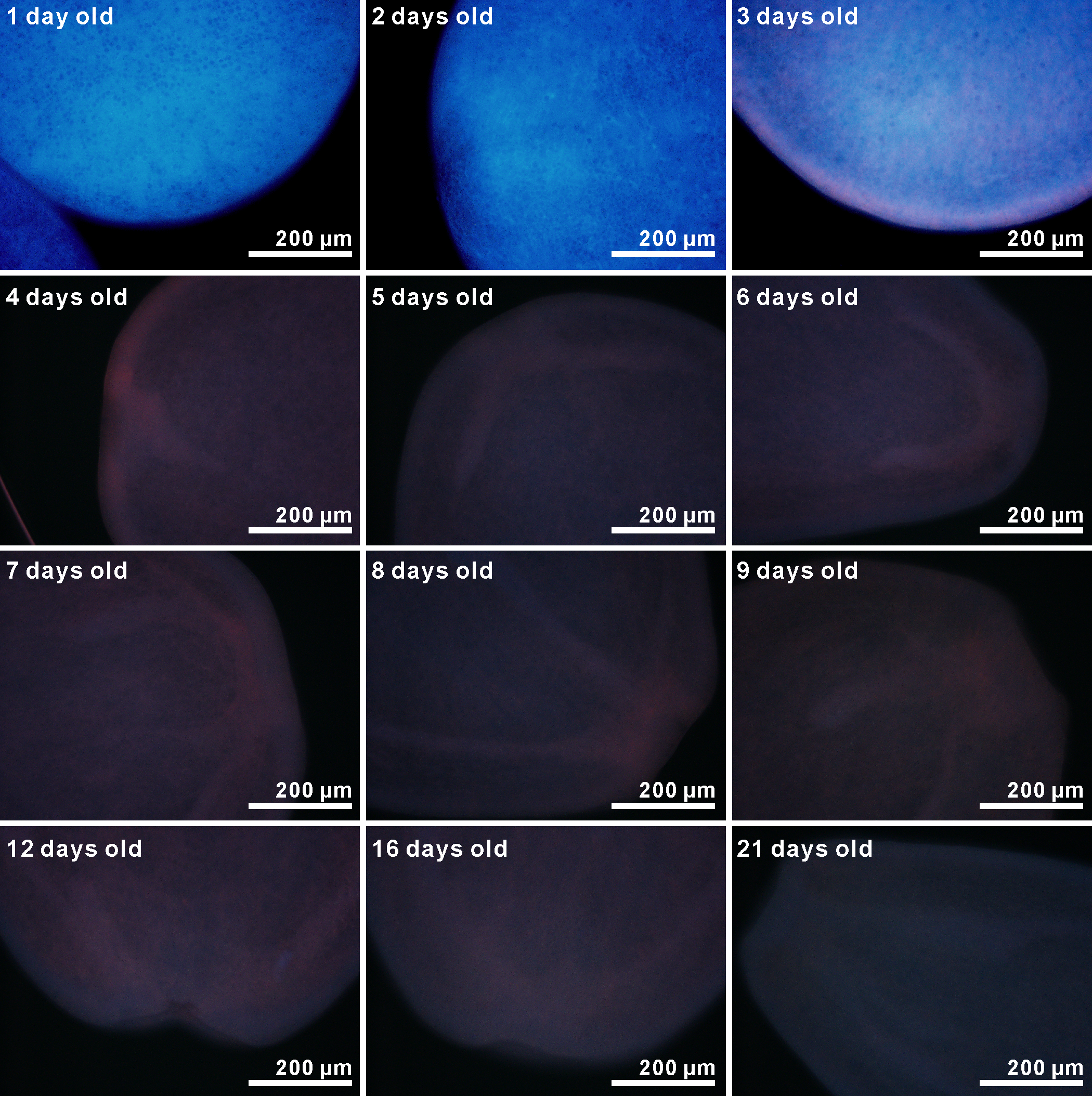

Supplement: S2 Fig — Micrographs with increased brightness are shown in Fig 5 in the main text. (TIF) [file pone.0252514.s002.tif]
