# Supplementary material for: Can Acropora tenuis larvae attract native Symbiodiniaceae cells by green fluorescence at the initial establishment of symbiosis?
Source: PLoS One. 2021 Jun 1;16(6):e0252514. doi: 10.1371/journal.pone.0252514 (PMC8168901; doi:10.1371/journal.pone.0252514)
Supplement: S1 Appendix — (DOCX) [file pone.0252514.s003.docx]

S1 Appendix

**Fluorescent protein-like proteins in *Acropora* *tenuis***

**Phylogenetic analysis**

The fluorescent protein-like protein-encoding genes were searched from the *Acropora* *tenuis* genome (BioProject ID: PRJDB8519; Shinzato et al., 2020) by using BLASTP (evalue = 1e^−5^) based on the *Acropora* *digitifera* fluorescence protein (Shinzato et al., 2012). All amino acid sequences were aligned with MAFFT (Katoh and Standley 2013) using the --auto option, and all gaps in the alignment were removed with TrimAL (Capella-Gutierrez, et al. 2009). Then, a maximum likelihood analysis was performed using the gap-free amino acid sequences (96 amino acids in length) from RAxML (Stamatakis 2014) with 100 bootstraps and the PROTGAMMAAUTO option (selected best model was WAG).


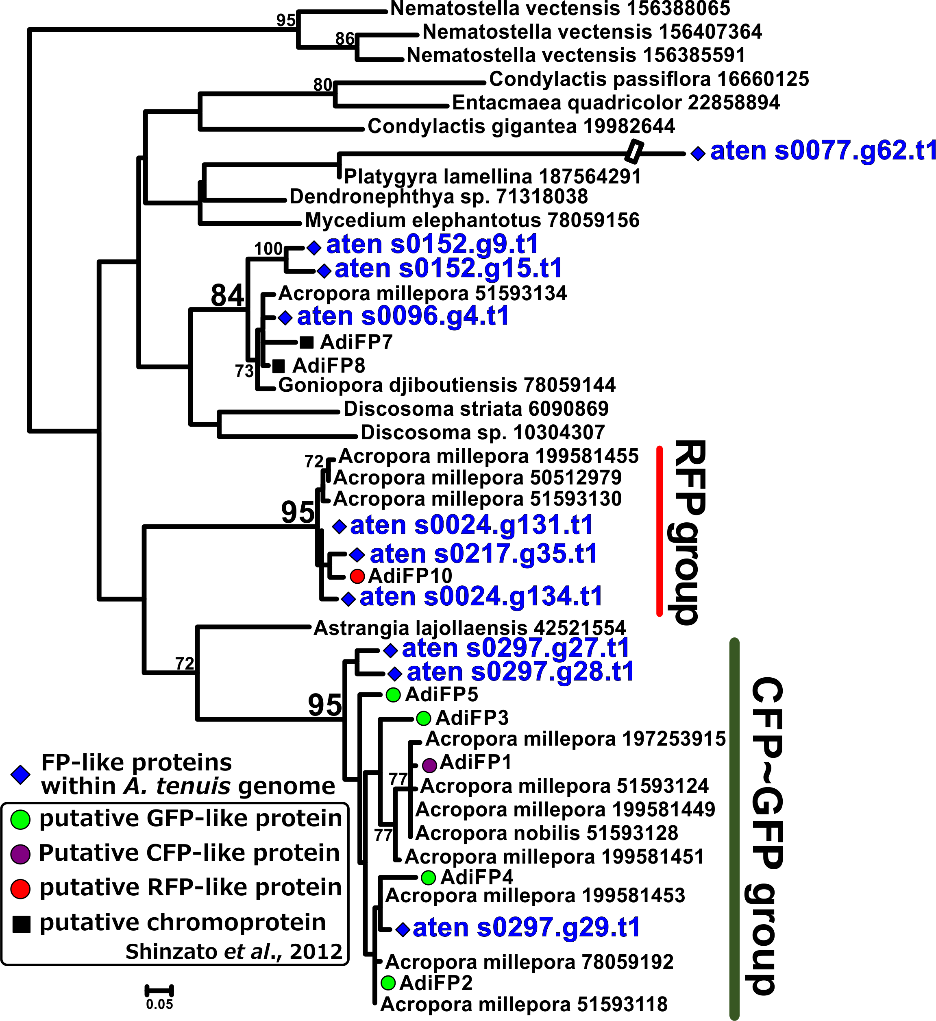


**Appendix S1 Fig. 1. Phylogenetic relationship of fluorescent protein-like proteins searched from the *A*. *tenuis* genome (blue text).** The color classes of *Acropora* *digitifera* fluorescent proteins reported by Shinzato et al., (2012) are also shown in the tree. Corresponding bootstrap proportions (≥70%) are shown by the branches.

**Protein analysis**

In this analysis, we prepared apo-symbiotic *A*. *tenuis* larvae from parental colonies that collected from Sekisei lagoon between Ishigaki and Iriomote islands in the southern part of Okinawa, Japan. Sampling of corals for research purposes was permitted by Okinawa Prefectural Government (No. 25-49). The tryptic digest was prepared using PTS solution (Masuda et al., 2009) with some modification. One hundred frozen planulae larvae at 16-day-old were crashed with freeze crasher AUTOMILL TK-AM7 (Tokken, inc, Chiba, Japan) at 1200 rpm for 90 sec. Next, 200 µL of PTS solutions was added, followed by sonication with Bioruptor (Cosmo Bio, Tokyo, Japan) for 15 min in ice water. The mixture was heated to 95°C for 10 min and quickly chilled on ice. The mixture was centrifuged at 15,000 × *g* for 10 min at 4°C, and the supernatant was transferred to a new PROKEEP tube (Fukae Kasei, Hyogo, Japan). After protein quantification by BCA protein assay, 50 µg of proteins were transferred to a new tube, reduced in 10 mM dithiothreitol, and alkylated in 50 mM iodoacetamide. After 4× dilution with 50 mM NH_4_HCO_3_, 0.5 µg proteomics-grade trypsin was added to the solution (MilliporeSigma, St. Louis, MO, USA), and then incubated at room temperature overnight. The tryptic digest was purified using GL-Tip SDB (GL Science, Tokyo, Japan). The purified peptides were analyzed with LC-MS/MS TripleTOF5600^+^ (SCIEX, Tokyo, Japan). The peptides were separated using a reverse-phase column Cadenza CD-C18 (Imtakt, Kyoto, Japan) preequilibrated with 0.1% TFA and 5% acetonitrile (MeCN). The peptides were separated using a MeCN gradient. The digested peptides were analyzed with data-dependent acquisition to identify the peptides. The obtained data was analyzed using Comet (Eng et al., 2012) in the trans proteomic pipeline (Deutsch et al., 2015) with the *A. tenuis* protein database based on the aforementioned *A*. *tenuis* genome.


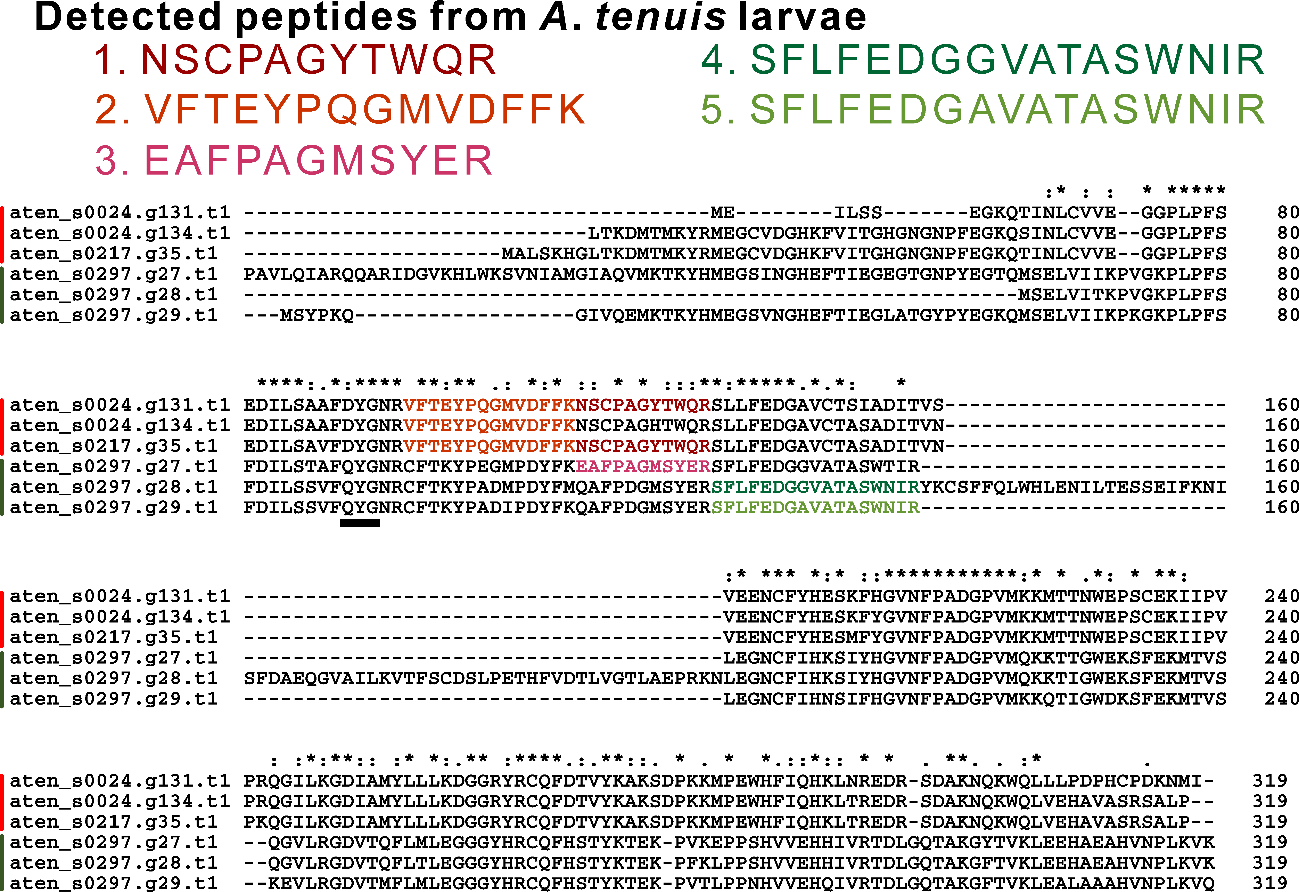


**Appendix S1 Fig. 2. Proteomic analysis of the larvae showed five peptides of GFP-like proteins, numbered 1 to 5.** These peptides were aligned to the several GFP-like proteins found from the *A. tenuis* genome (Appendix S1 Fig. 1) using Clustal X 2.1 (Larkin et al., 2007). The red bar indicates the RFP group and the green bar indicates the CFP~GFP group in Appendix S1 Fig. 1. The colored amino acid sequences correspond to peptides detected. The black bar indicates the chromophore. The symbols *, :, ., indicate identical, conserved, and similar amino acids, respectively.

**References for S1 Appendix**

Shinzato C, Khalturin K, Inoue J, Zayasu Y, Kanda M, Kawamitsu M, Yoshioka Y, Yamashita H, Suzuki G, Satoh N (2020) Eighteen coral genomes reveal the evolutionary origin of *Acropora* strategies to accommodate environmental changes. Mol Biol Evol 38: 16–30. https://doi.org/10.1093/molbev/msaa216

Shinzato C, Shoguchi E, Tanaka M, Satoh N (2012) Fluorescent protein candidate genes in the coral *Acropora* *digitifera* Genome. Zool Sci 29: 260–264.

Katoh K, Standley DM (2013) MAFFT Multiple sequence alignment software version 7: Improvements in performance and usability. Mol Biol Evol 30: 772–780.

Capella-Gutiérrez S, Silla-Martínez JM, Gabaldón T (2009) TrimAl: a tool for automated alignment trimming in large-scale phylogenetic analyses. Bioinformatics 25: 1972–1973.

Stamatakis A (2014) RAxML version 8: a tool for phylogenetic analysis and post-analysis of large phylogenies. Bioinformatics 30: 1312–1313.

Masuda T, Saito N, Tomita M, Ishihama Y (2009) Unbiased quantitation of Escherichia coli membrane proteome using phase transfer surfactants. Mol Cell Proteomics 8: 2770–2777. https://dx.doi.org/10.1074/mcp.m900240-mcp200

Eng J, Jahan T, Hoopmann M (2012) Comet: an open-source MS/MS sequence database search tool. Proteomics 13: 22–24. https://dx.doi.org/10.1002/pmic.201200439

Deutsch E, Mendoza L, Shteynberg D, Slagel J, Sun Z, Moritz R (2015) Trans-Proteomic Pipeline, a standardized data processing pipeline for large-scale reproducible proteomics informatics. Prot Clin Appl 9: 745–754. <https://dx.doi.org/10.1002/prca.201400164>

Larkin MA, Blackshields G, Brown NP, Chenna R, McGettigan PA, McWilliam H, Valentin F, Wallace IM, Wilm A, Lopez R, Thompson JD, Gibson TJ, Higgins DG (2007) Clustal W and Clustal X version 2.0. Bioinformatics 23:2947–2948
