# Supplementary material for: Can Acropora tenuis larvae attract native Symbiodiniaceae cells by green fluorescence at the initial establishment of symbiosis?
Source: PLoS One. 2021 Jun 1;16(6):e0252514. doi: 10.1371/journal.pone.0252514 (PMC8168901; doi:10.1371/journal.pone.0252514)
Supplement: S2 Appendix — (DOCX) [file pone.0252514.s004.docx]

S2 Appendix

**Greyscale images of separated color channels (red, green, and blue) of Figs 3 and 5.**

The original RGB colored Figs. 3 and 5 in the main texit are fluorescent micrographs of *A*. *tenuis* larvae taken under blue-violet excitation (Ex. 400–440 nm, Em. ≥475 nm) and UV-A excitation (Ex. 330–385 nm, Em. ≥420 nm), respectively. These colored micrographes were separated for each color channel (red, green, and blue) using Image J (Rasband, W.S., ImageJ, U. S. National Institutes of Health, Bethesda, Maryland, USA, https://imagej.nih.gov/ij/, 1997-2018). It should be noted that the original colored micrographs of larvae aged 1–3 days were taken with an exposure time of 500 ms, while those of larvae older than 4 days were taken with an exposure time of 50 ms and without any digital black balances. Thus, micrographs of larvae aged 1–3 days and those older than 4 days are slightly different in color.


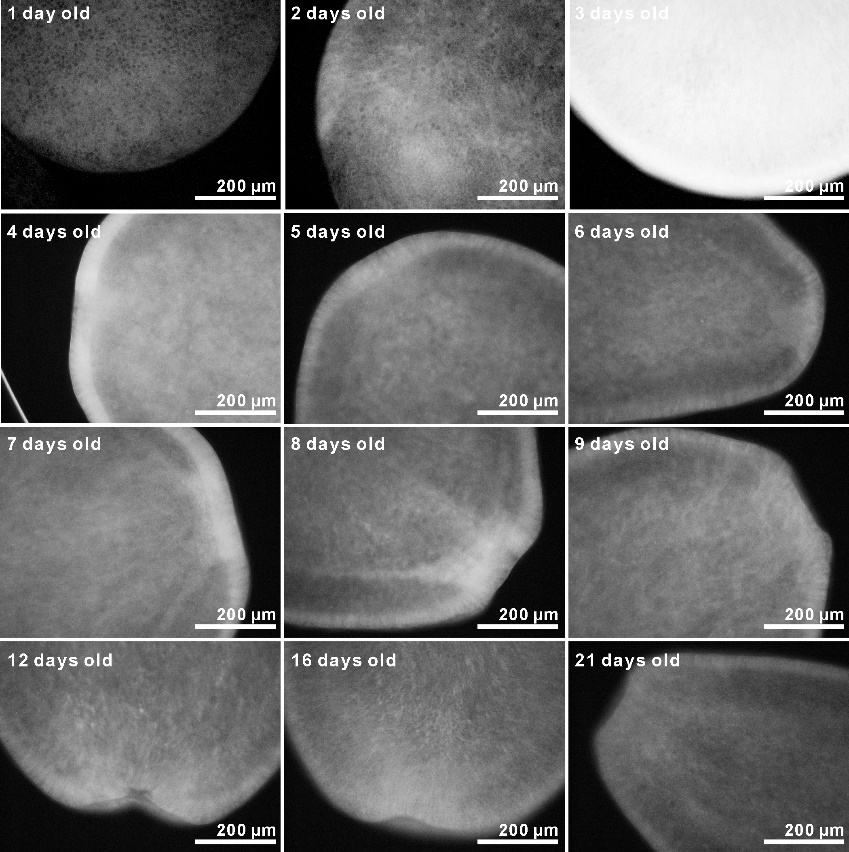


**Red channel of Fig. 3**


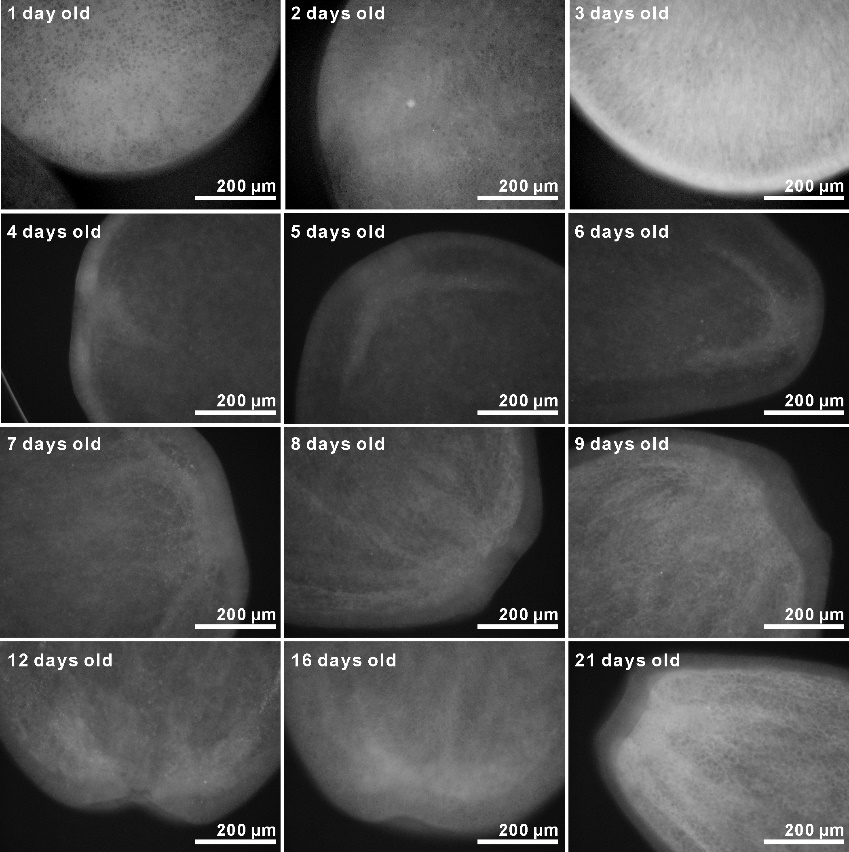


**Green channel of Fig. 3**





**Blue channel of Fig. 3**


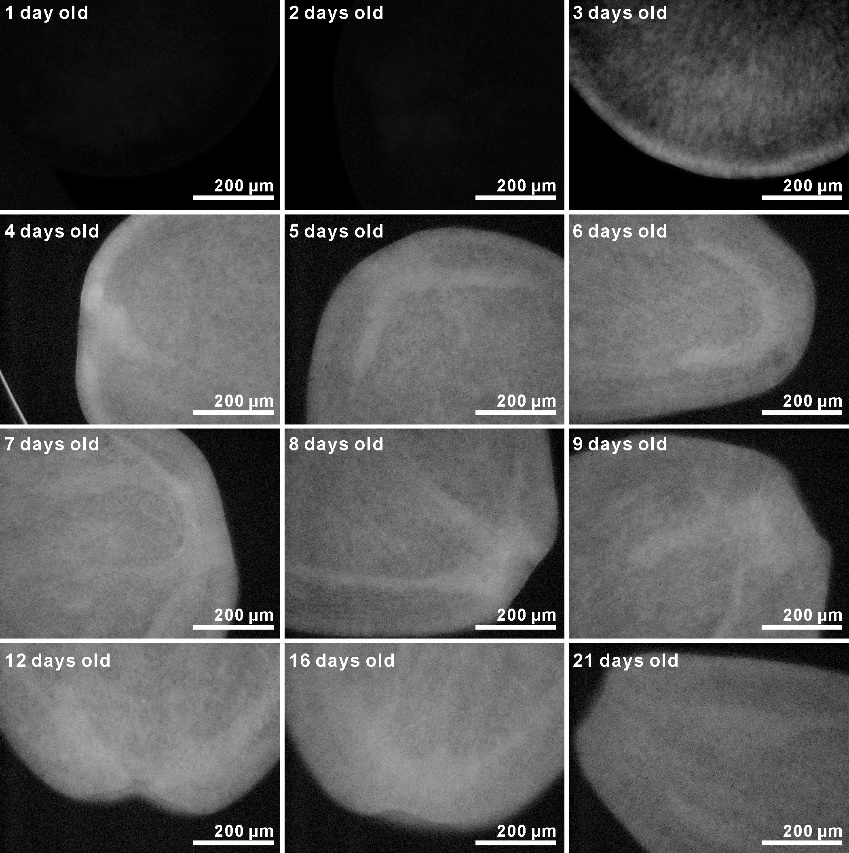


**Red channel of Fig. 5**


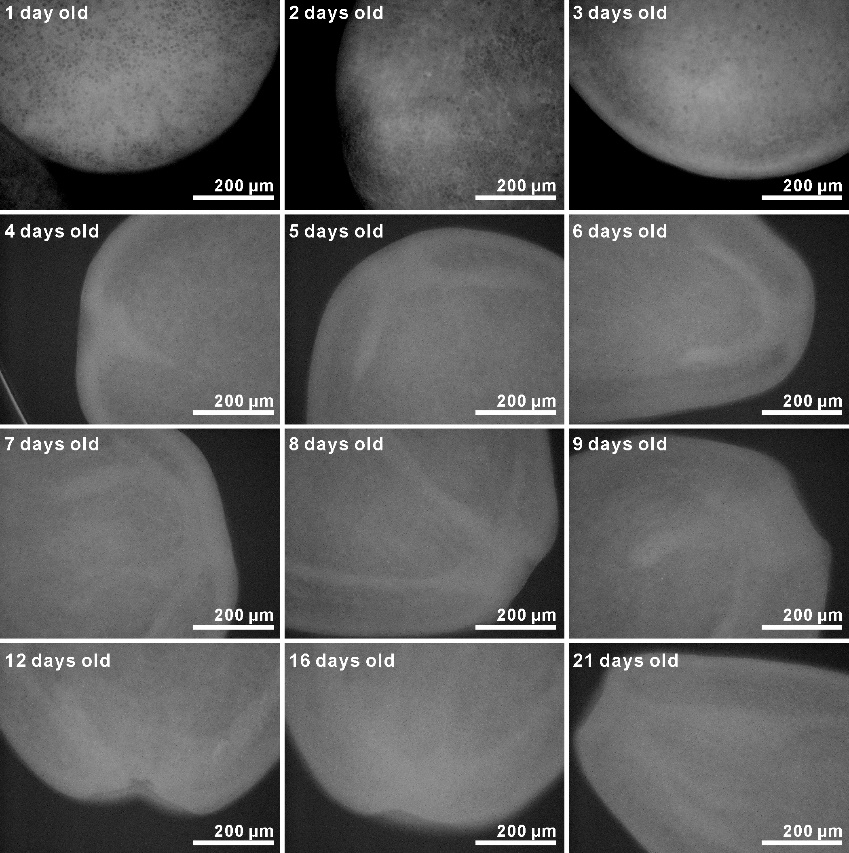


**Green channel of Fig. 5**


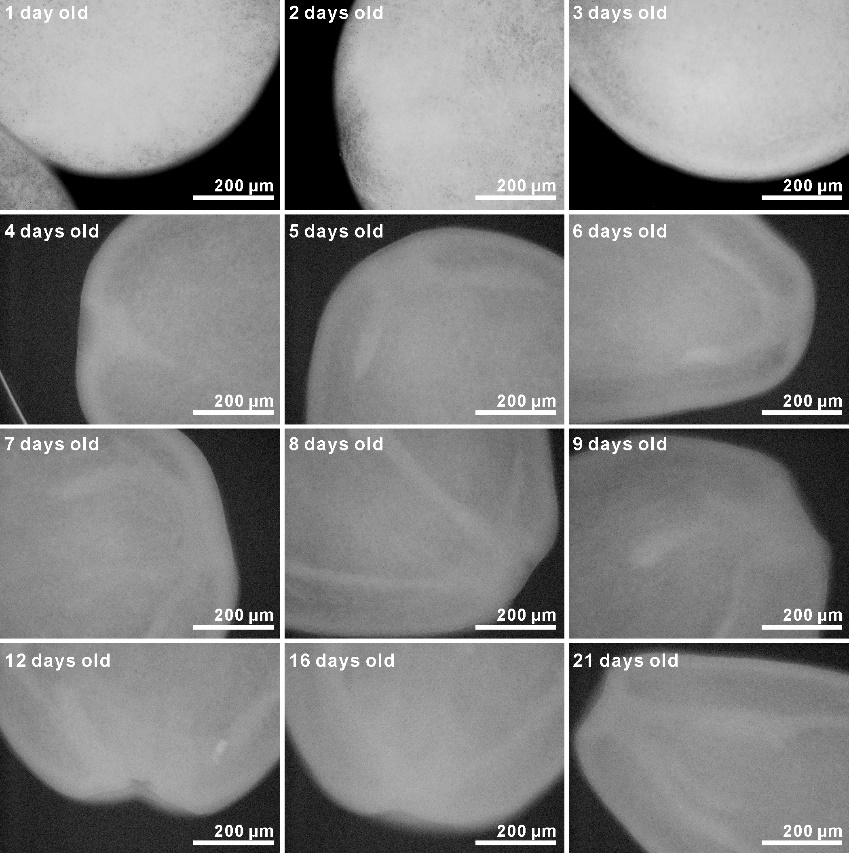


**Blue channel of Fig. 5**
