# Supplementary material for: Can Acropora tenuis larvae attract native Symbiodiniaceae cells by green fluorescence at the initial establishment of symbiosis?
Source: PLoS One. 2021 Jun 1;16(6):e0252514. doi: 10.1371/journal.pone.0252514 (PMC8168901; doi:10.1371/journal.pone.0252514)
Supplement: S3 Appendix — (DOCX) [file pone.0252514.s005.docx]

S3 Appendix

**Fluorescent micrographs and** **fluorescence spectra of faint orange fluorescence larvae (greenish larvae)**

**Fluorescent micrographs**


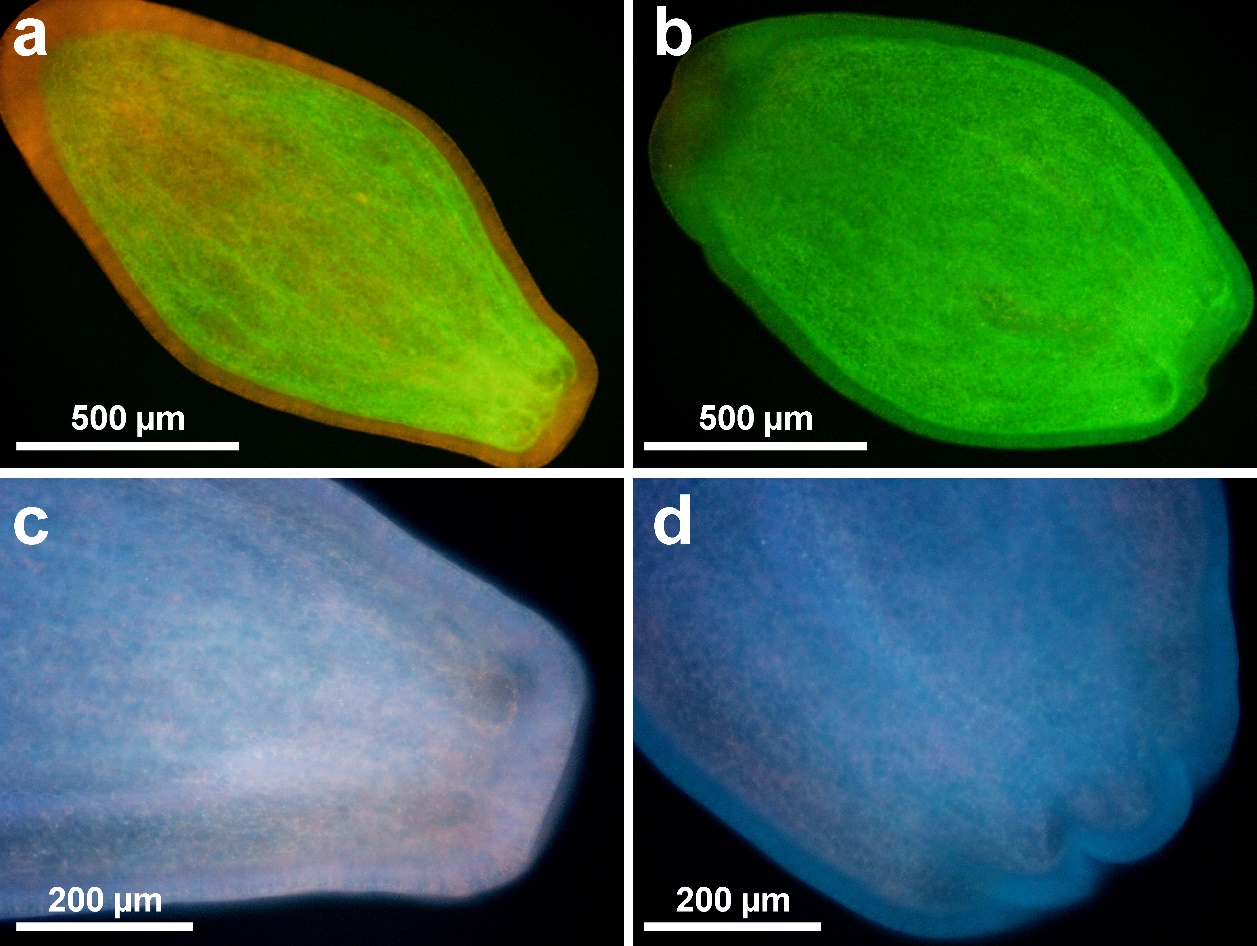
During our observations, we occasionally found faint orange fluorescence larvae. These larvae were visually greenish under a fluorescent microscope compared larvae with orange fluorescence. At larval age of 21 d, larvae with orange fluorescence and faint orange fluorescence larvae (greenish larvae) were observed under an epifluorescence microscope (BX50, Olympus, Tokyo, Japan). Fluorescence filter cubes were U-MWBV2 (Ex. 400–440 nm, Em. ≥475 nm; blue-violet excitation) and U-MWU (Ex. 330–385 nm, Em. ≥420 nm; UV-A excitation). Fluorescence micrographs of the larvae were taken using an ultrasensitive charge-coupled device camera (DP-73, Olympus, Tokyo, Japan).

**Appendix S3 Fig 1. Fluorescent micrographs of 21-days-old *A*. *tenuis* larvae taken under blue-violet excitation (a, b) and UV-A excitation (c, d).** a, c) Larvae with orange fluorescence. b, d) faint orange fluorescence (greenish) larvae.

**Fluorescence spectra of faint orange fluorescence larvae (greenish larvae)**

**

**Fluorescence spectra of greenish larvae at 4, 6, 7, 8, 12, 16, and 21 days-old were measured using a Photonic Multi-channel Analyzer (PMA-C7473, Hamamatsu Photonics K.K., Shizuoka, Japan) set at on the camera port of the microscope. The settings of measurements were same as in the main text. Standardization using fluorescence reference slides was not conducted.

**Appendix S3 Fig 2. Fluorescence spectrum of greenish *A*. *tenuis* larvae under blue-violet excitation (Ex. 400–440 nm, Em.** ≥**475 nm).** Peak wavelength at which the maximum intensity was recorded is shown in each graph. The y-axis values for the 21 days-old larvae are different from the others.

**

Appendix S3 Fig 3. Fluorescence spectrum of greenish *A*. *tenuis* larvae under UV-A excitation (Ex. 330–385 nm, Em.** ≥**420 nm).** Peak wavelength at which the maximum intensity was recorded is shown in each graph.
