# Supplementary material for: Can Acropora tenuis larvae attract native Symbiodiniaceae cells by green fluorescence at the initial establishment of symbiosis?
Source: PLoS One. 2021 Jun 1;16(6):e0252514. doi: 10.1371/journal.pone.0252514 (PMC8168901; doi:10.1371/journal.pone.0252514)
Supplement: S4 Appendix — (DOCX) [file pone.0252514.s006.docx]

S4 Appendix

**Acquisition of native Symbiodiniaceae cells by *A*. *tenuis* larvae under blue LED light**

The experimental procedurs were same as in “Acquisition of Symbiodiniaceae cells by A. tenuis larvae under red LED light” part in the main text. Namely, we prepared *A*. *tenuis* larvae following to Suzuki (2020). Ten parental colonies were collected from Sekisei lagoon, and sampling of parental corals was permitted by the Okinawa Prefectural Government for research use (No. 24-54). Five individual 6-day-old apo-symbiotic larvae were put into each of six 100 mL glass cups (columnar form; inner diameter = 47 mm, height = 54 mm) with 50 mL of 0.4-µm-filtered seawater. Fifty cells of cultured strains AJIS2-C2 (*S*. *microadriaticum*) or CCMP 2556 (*D*. *trenchii*) were added to the cups in triplicate. This cell density (1000 cells/L) is similar to that in the natural reef environment Yamashita et al., (2013), and even at such a low cell density, *A*. *tenuis* larvae can acquire these native symbionts Yamashita et al., (2014). All experimental cups were then placed under a blue LED light source (CWL; 470 nm, FWHM; 30 nm, EYELA TOKYO RIKAKIKAI CO., LTD, Tokyo, Japan) from 07:00 to 20:00. Subsequently, all larvae, without fixation, were observed under an epifluorescence microscope (BX50; filter cube U-MNV; Ex. 400−410 nm, Em. ≥455 nm longpass) to count the acquired algal cells within the larval body, as previously described by Yamashita et al., (2014).

We conducted a likelihood ratio test (LRT) based on a generalized linear model with mixed effects (GLMM) for analysis of the infection test under blue LED light. The statistical tests were performed using R versinon 3.6.3 (R Core Team 2020) and glmmTMB package, version 1.0.1 (Brooks et al., 2017). The percentage of Symbiodiniaceae cells acquired larvae (= infection rates) was assumed to follow a binomial distribution, and the link function was logit. The explanatory variable was the Symbiodiniaceae species (*S*. *microadriaticum* or *D*. *trenchii*); the random effect was assumed to vary among the experimental glass cups. For the acquired symbiont cell densities within the exposed individual larva, the statistical test assumed a Poisson distribution of cell numbers and adopted a log link function. Other components were the same as the model for infection rates. LRT was conducted between these models and the null model, excluding the explanatory variable (supplied symbiont species) using the anova function in R. In the present study, *p*-values < 0.05 were considered to be statistically significant.

We observed 15 *A*. *tenuis* larvae supplied with either *S*. *microadriaticum* or *D*. *trenchii* (5 larvae / cup × three cups each). The percentages of *S*. *microadriaticum* infected larvae in each cups under blue LED light were 40%, 40%, and 40%, and the percentages of *D*. *trenchii* infected larvae under blue LED light were 80%, 80%, and 40% (S4 Appendix Fig1). The percentage of Symbiodiniaceae cell infected larvae was not significantly changed between symbiont species (Δdeviance = 2.1696, Δdf = 1, *p* = 0.1408). Acquired *S*. *microadriaticum* cell numbers within each larva under blue LED light was 1.2 ± 0.17 cells/larva. Acquired *D*. *trenchii* cell number in larva was 2.8 ± 0.44 cells/larva. Numbers of symbiont cells acquired by larvae significantly differed depending on symbiont species (Δdeviance = 9.3762, Δdf = 1, *p* = 0.002198).


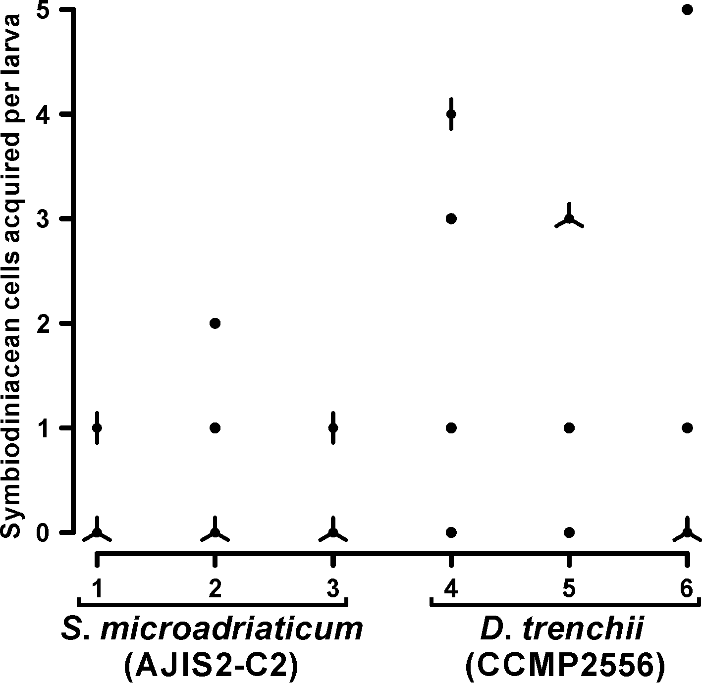


**Appendix S4 Fig 1. The algal numbers within larvae under blue LED light source.** Acquired cell numbers are shown in a sunflower plot for each experimental cup separately. Number of leaves (petals) indicating that individual larvae harbored the same numbers of symbiont cells. Five individual 6-day-old larvae were observed in each experimental cup.

**References for Appendix**

Suzuki G (2020) Optimization of a spawning-induction protocol for the prediction of natural coral spawning. Fish Sci 86: 665–671.

Yamashita H, Suzuki G, Hayashibara T, Koike K (2013) *Acropora* recruits harbor ‘‘rare’’ *Symbiodinium* in the environmental pool. Coral Reefs 32: 355–366.

Yamashita H, Suzuki G, Kai S, Hayashibara T, Koike K (2014) Establishment of coral-algal symbiosis requires attraction and selection. PLoS ONE 9: e97003.

R Core Team (2020). R: A language and environment for statistical computing. R Foundation for Statistical Computing, Vienna, Austria. URL <https://www.R-project.org/>.

Brooks ME, Kristensen K, van Benthem KJ, Magnusson A, Berg CW, Nielsen A, Skaug HJ, Maechler M, Bolker BM (2017) glmmTMB balances speed and flexibility among packages for zero-inflated generalized linear mixed modeling. The R Journal 9: 378–400.
