# Supplementary material for: Can Acropora tenuis larvae attract native Symbiodiniaceae cells by green fluorescence at the initial establishment of symbiosis?
Source: PLoS One. 2021 Jun 1;16(6):e0252514. doi: 10.1371/journal.pone.0252514 (PMC8168901; doi:10.1371/journal.pone.0252514)
Supplement: S5 Appendix — (DOCX) [file pone.0252514.s007.docx]

S5 Appendix

**Fluorescence spectra of three adult *Acropora tenuis* colonies**

Three adult *A*. *tenuis* colonies kept in the running natural seawater tank were used for observation. These colonies were collected from Sekisei lagoon between Ishigaki and Iriomote island in the southern part of Okinawa, Japan. Sampling of corals was permitted by the Okinawa Prefectural Government for research use (No. 28-76). The small pieces of the colonies were observed under the an epifluorescent microscope (BX50, 4× objective lens, Olympus, Tokyo, Japan). The fluorescence filter cubes were U-MWU (Ex. 330–385 nm, Em. ≥420 nm; UV-A excitation) and U-MWBV2 (Ex. 400–440 nm, Em. ≥475 nm; blue-violet excitation). The fluorescent spectrum was measured by the PMA-C7473 (Hamamatsu Photonics K.K., Shizuoka, Japan), and the exposure time was set at 5000 ms). Additionally, the background away from the pieces of colonies was measured. Standardization using fluorescence reference slides was not conducted.


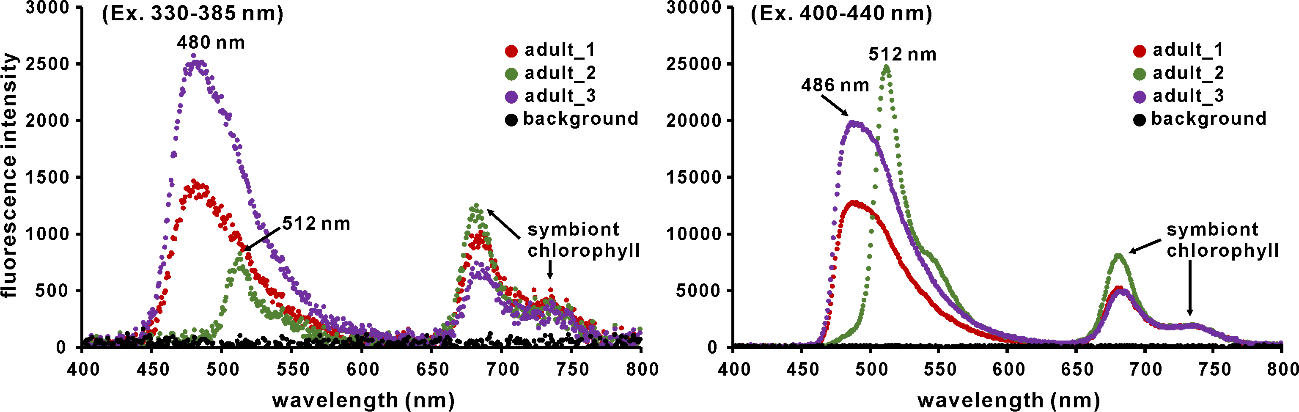


**Appendix S5 Fig 1. Fluorescence spectrum of three adult *A*. *tenuis* colonies.** In the adult colonies, orange fluorescence was not observed; however, blue (480–486 nm) and green (512 nm with about 550 nm shoulder) fluorescence was observed. The fluorescent pattern was completely different between adult_2 and adult_1,3.
