# Supplementary material for: Can Acropora tenuis larvae attract native Symbiodiniaceae cells by green fluorescence at the initial establishment of symbiosis?
Source: PLoS One. 2021 Jun 1;16(6):e0252514. doi: 10.1371/journal.pone.0252514 (PMC8168901; doi:10.1371/journal.pone.0252514)
Supplement: Attachment — Submitted filename: PONE-D-20-25327_R1_Reviewer_Comments.pdf [file pone.0252514.s013.pdf]

Major general comments:

1. I still would like to see measured light intensities for the first experiment, in the simplest set up just the different filters between the Xenon lamp and the sensor.

2. There are still a few technical details in the Materials & Methods section which should be added (see the specific comments).

3. For presenting the data on the phototaxis spectra of the different algal strains, I would recommend indicating the strains with statistically significant responses in the figure. Is there any way to obtain wavelength-specific significances of the responses from the analysis? That would make the graphical representation easy (asterisk above the bars) and also give another layer of useful information as the paper aims at untangling the responses to the different light qualities. Additionally, I would suggest including the details from S1 Fig in the results paragraph to point to the different wavelength responses, the easiest way to do that might be by just giving the wavelength ranges with a positive and a negative response. Also, I would recommend using the same y-axis scale for all graphs in S1 Fig. to make it more easy to compare them visually.

4. My concern about the fluorescence images of the 1 to 4-day old larvae (Fig. 3 and Fig. 5) was not about the absence of larval fluorescence, it was mainly about the absence of any (!) background noise (compare the background for the older larvae vs. that of any of the first four days) and the absence of finer graduations in the 3 and 4-day images under violet-blue excitation where the intensity between areas which show fluorescence drops directly to 0 (see the included line scan from 4 vs. 5 days). This indicates to me that the histogram of the raw images was somehow trimmed (by adjusting brightness & contrast or applying background subtraction etc.) before converting it to the final images. This does not change the validity of the results and is merely a technical issue. But I think it is important to follow good practices in image processing and presentation and things like this can easily happen by some hidden default settings in image processing software (e.g., after re-installation of the software), especially when converting from 16-bit to 8-bit or RGB images.

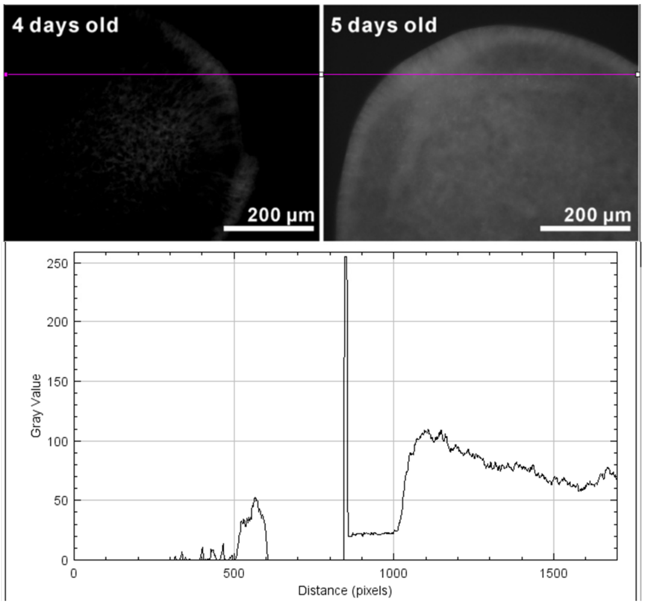

5. I strongly oppose the removal of the infection results under 470 nm light. I feel that the results for at least *S. microadriaticum* are interesting as they show a positive effect (or non-negative) effect for the blue excitation. The action spectrum for *S. microadriaticum* is pretty clear in that green fluorescence is rather unlikely to cause this effect but looking at the larval fluorescence spectra under similar excitation, it seems plausible to me that the orange-red fluorescence might have an effect. I think this should be at least discussed although I would prefer to see an experiment on the action spectra of at least *S. microadriaticum* and *D. trenchii* with one or two filters covering those longer wavelengths and maybe one of the previous ones as a reference. I also still would like to see one more wavelength around 440-450 nm. In general, this experiment still needs some more explanation in the text to link it better to the previous experiments and an infection experiment under UV-A light should be at least mentioned in the discussion. Furthermore, I would strongly recommend linking the infection data to the ones (in white light I think?) from Yamashita et al., 2014, especially since no dark or white light controls were performed in the current manuscript.

<u>Minor general comments:</u>

1. For some references, DOIs are given and for some not. I would suggest being consistent (and in accordance with the PLoS ONE requirements).

2. In the supplemental datasets S2 and S3, I would highly recommend trimming the data to include only the wavelengths within the filter specs (e.g., starting only at 475 nm for blue-violet excitation and 420 nm for UV-A excitation) or, probably better, to the range shown in the graphs in Fig. 4 and Fig. 6. The authors should also consider setting negative values to zero as there cannot be less light than none.

3. I appreciate the effort of the authors to include the greyscale images as S2 and S4 Figs., but I should have been more clear; I thought of the supplemental figures to show the different color channels (red, green and for S4 Fig also blue) as greyscale separately, side-by-side. After going back to the Materials & Methods and looking up the Olympus DP-73 camera and the filter cubes, I realized that this is an RGB camera and the emission filters were longpass filters. This does not affect the above comment, but I would strongly suggest including this information in the respective Material & Methods section.

<u>Specific comments:</u>

L39-41: "…mechanisms may exist to facilitate encounter with suitable symbiont cells, by new coral generations. Green fluorescence in corals has been proposed as a mechanism to attract these cells."

- I would suggest simplifying these sentences and to introduce phototaxis in the second sentence e.g.: "…mechanisms may exist by which new coral generations attract suitable symbionts. Phototaxis of suitable symbionts towards green fluorescence in corals has been proposed as such a mechanism."

L66: "However, early life stages of the majority of corals that use <u>spawn-gamete reproduction</u>…"

- Might this be a direct word-by-word translation from Japanese? I think the usual English expression would be “broadcast spawners” (corals that release their gametes into the water column where they fertilize and then develop in contrast to brooding spawners where only sperm is released, and eggs are fertilized and develop within the parent colony and often inherit the symbionts vertically).
  If that assumption is correct, the sentence could be changed to e.g.: “However, early life stages of the majority of broadcast-spawning corals…”

L102: “…several Symbiodiniaceae cultured strains…”

- “…several cultured Symbiodiniaceae strains…”

L129-130: “During the early to mid-logarithmic proliferation phase of each strains…”

- “During the early to mid-logarithmic proliferation phase of each strain…”

L130: “(Φ 25 mm)”

- This is the Greek letter phi, there is a specific symbol for the diameter in the Unicode tables (U+2300; ⌀, unfortunately not available in most common fonts; but U+00F8, ø, should be a valid substitute in most fonts).

L177: “epifluorescent microscope”

- Must be “epifluorescence microscope”.

L178-179: “Fluorescence filter cubes were U-MWBV2 (Ex. 400–440 nm, Em. 475 nm; blue-violet excitation) and U-MWU (Ex. 330–385 nm, Em. 420 nm; UV-A excitation).”

- I would suggest clarifying that the emission filters were longpass filters in contrast to the excitation bandpass filters by i.e.: “Fluorescence filter cubes were U-MWBV2 (Ex. 400–440 nm bandpass, Em. ≥ 475 nm longpass; blue-violet excitation) and U-MWU (Ex. 330–385 nm bandpass, Em. ≥ 420 nm longpass; UV-A excitation).” In addition, I would suggest adding the information that the camera is an RGB-color camera to line 180-181 (see also minor general comment 3. on Figs. S2 and S4).

L183-185: “…set at on the camera port of the microscope.”

- Maybe change to: “…attached to the camera port of the microscope.”

L194: “…cause saturation of PMA under with settings used.”

- “…cause saturation of the PMA with the used settings.”

L211: “…red LED…”

- This occurs a number of times, I would recommend writing “red LED light”.

L225: “…epifluorescent microscope (BX50; 400−410 nm excitation)…”

- This must be “epifluorescence microscope”. Also please add the emission filter properties.

L284: “Fig 2. The phototaxis patterns s of Symbiodiniaceae cultured strains.”

- I would suggest “Fig 2. The phototaxis patterns of cultured Symbiodiniaceae strains.”

L284-286: “The 100% line indicates that initial cell density and cell density after light irradiation if not changed.”

- This sentence needs rephrasing. I would suggest something like “The 100% line indicates constant cell densities before and after irradiation.”

L296-298: “Although transmittance between 455 nm and 478 nm was less than 50% owing to characteristics of the emission filter, a shoulder was found between 467 nm and 507 nm (Fig 4).”

- Do the authors have access to the exact transmission characteristics of the filter so that it would be possible to correct the data for filter transmittance before plotting?

L313-314: “Owing to the characteristics of the emission filter, transmittance between 313 nm and 420 nm was less than 50%.”

- Same as previous comment.

L315: “Fluorescence intensities under UV-A were overall lower than under blue-violet.”

- “Fluorescence intensities under UV-A excitation were overall lower than under blue-violet excitation.”

L321-322: “…are found in *A. tenuis* genome (S1 Appendix). These six-fluorescent…”

- “…are found in the *A. tenuis* genome (S1 Appendix). These six fluorescent…”

L329: “…Em. 375 nm).”

- “…Em. ≥375 nm).”

L333: “…Em. 375 nm).”

- “…Em. ≥375 nm).”

L339: “…Em. 420 nm).”

- “…Em. ≥420 nm).”

L344: “…Em. 420 nm).”

- “…Em. ≥420 nm).”

L358: “…significantly differed depended…”

- “…significantly differed dependent…”

L376-377: "Native symbiont species *S. microadriaticum* cultured strain…"

- I would suggest a change to e.g., "Cultured algae of the native symbiont *S. microadriaticum*…"

L378-379: "A similar trend was also found in free-living species *S. natans* cultured strain…"

- Change to e.g. "A similar trend was also found in cultures of the free-living species *S. natans*…"

L381: "Although in the present study a shorter wavelength was not observed…"

- This sentence needs a bit more explanation. I think the authors mean that no phototaxis was observed at lower wavelengths for the strains used, but this is not directly clear from reading this sentence and I feel that it would also help the reader to very briefly, with half a sentence, describe the findings from the reference given here.

L409: "A simple compare"

- "A simple comparison"

L411: "…with that of reported by…"

- "…with that reported by…"

L449-450: "The light environment in these shaded spots is thought mainly scattered and reflected light rather than direct sunlight."

- "The light environment in these shaded spots is thought to consist of mainly scattered and reflected light rather than direct sunlight."

L458: "...infection test was conducted under red LED light."

- "...infection tests were conducted under red LED light."

L469: "…cells of *S. microadriaticum* cultured strain…"

- "…cells from *S. microadriaticum* cultures…"

L474: "Although a longer wavelength was not observed…"

- Just a bit more precise: "Although phototaxis response to a longer wavelength than 570 nm was not assessed…"

L480-481: "Thus, various spatial scales might exist for the distance between apo-symbiotic corals and their native symbionts according to the coral life stage."

- I don't understand this sentence.

L483: "…however, it is unclear these attractants are effective at longer distance."

- "…however, it is unclear if these attractants are effective at longer distance."

L494: "The peak wavelength…"

- "At the peak wavelength…"

L516-517: "If they are within shaded locations on substrates where larvae settle, these attractions might be possible in the field."

- Is it not much more likely that larvae acquire their symbionts in the water column while they are swimming and where the light intensity is high enough to reasonably excite the fluorescent proteins before settlement?

L521: "…are needed."

- "…is needed."

Filename: "new_S2_dataset_fluorescence spectra under biolet-bule.xlsx"

- "new_S2_dataset_fluorescence spectra under violet-blue.xlsx"

Appendix S1 Fig. 2:

- From the figure legend, it is not fully clear to me, which exact peptide sequences are meant by the numbers for 1,3 as well as 4,5 where the sequences slightly differ within the RFP or CFP~GFP groups in the indicated regions.
- Additionally, I would suggest indicating the amino acid triplet which gives rise to the chromophore and maybe adding a secondary structure prediction (maybe with a well-studied FP, e.g. AvGFP, as reference).
- And just a minor thing: I believe it must be "conserved" and not "conservative" in the last line of the legend.
