# Supplementary material for: Can Acropora tenuis larvae attract native Symbiodiniaceae cells by green fluorescence at the initial establishment of symbiosis?
Source: PLoS One. 2021 Jun 1;16(6):e0252514. doi: 10.1371/journal.pone.0252514 (PMC8168901; doi:10.1371/journal.pone.0252514)
Supplement: Attachment — Submitted filename: Reviewer_Comments.pdf [file pone.0252514.s015.pdf]

Major general comments:

1. **L277-295:** Looking at the graphs in Fig. 2, I think the authors need to discuss here not only the statistical significance but also the possible biological one. Especially the differences for *B. minutum* are so minimal that I would be really surprised if they had any relevance for attraction (even though they are statistically significant). The opposite seems true for *D. trenchii*, where the variance between replicates prohibits any statistical significance but there might be an actual underlying pattern. In my opinion, this should be mentioned in one or two additional sentences.

Specific comments:

L48: "Although the larvae fluoresced with green and broad orange fluorescence…"

- I would suggest changing this to "Although the larvae exhibited green and broad orange fluorescence…"

L58-61: "Thus, attraction mechanisms other than visible fluorescence may exist, at least for *D. trenchii* . Our results suggest that attraction mechanisms other than green fluorescence could exist, a topic which requires further investigation and discussion."

- The two sentences are mostly redundant. I would recommend collapsing them into one sentence.

L81: "…some mechanism likely exist**s**…"

- I suggest changing this to "…some mechanism**s** likely exist…" to account for the possibility of multiple parallel mechanisms for attraction.

L82: "Laboratory experiments show that some Symbiodiniaceae cultured strains…"

- Should be "Laboratory experiments show that some cultured Symbiodiniaceae strains…"

L87-88: "…juvenile polyps of soft coral *Heteroxenia fscescens*."

- Better: "…juvenile polyps of **the** soft coral *Heteroxenia f**u**scescens*."

L88-89: "…green fluorescence protein (GFP)…"

- "…green fluorescen**t** protein (GFP)…"

L222: "…following Suzuki [25]."

- I think, this must be "…following Suzuki**, 2020** [25]."

L240: "…we used generalized additive model…"

- This should be "…we used **the** generalized additive model…"

L305-309:

- I think, this information is important but is sufficient to include only in the figure legends. Therefore, I would recommend to completely delete this paragraph.

L313: "…orange fluorescence was observed after 2-day-old larvae."

- Better: "…orange fluorescence was observed after 2 days."

L338-339: "…even in the 1-day-old coral. The orange fluorescence was also observed more than the 3-day-old larvae."

- I would suggest writing: "…even in the 1-day-old larvae. Orange fluorescence was observed in larvae older than 3 days."

L349: "…however, a 522 nm green fluorescence peak…"

- "…however, the 522 nm green fluorescence peak…"

L351-354:

- I highly recommend moving this part on the proteomics to the beginning of the section (before L310).

L359: "Micrographs 1 to 3-day-old larvae…"

- "Micrographs **of** 1 to 3-day-old larvae…"

L425-426: "…, and calculations based on the thickness of each layer, it is ideal for reflection of light with wavelengths of 320–520 nm [23]."

- I would recommend changing this to: "…, and calculations based on the thickness of each layer **indicate that** it is ideal for reflection of light with wavelengths of 320–520 nm [23]."

L440: "…*E. aspera* coral emits…"

- "…*E. aspera* corals emit…"

L441: "cultured symbiodiniacean strain **"**OTcH-1**"** shows"

- Remove the quotation marks.

L458: "A broad "orange" fluorescence"

- Remove the quotation marks.

L502-504: "However, genus *Durusdinium* including *D. trenchii* **were** often found within **the** *Acropora* larvae/juveniles [10,13,14,46,47]."

- I would suggest “However, genus *Durusdinium* including *D. trenchii* **is** often found within *Acropora* larvae/juveniles [10,13,14,46,47].”

L527-528: “A chemotaxis experiment using soft coral *H. fuscescens* polyps…”

- Better: “A chemotaxis experiment using polyps of the soft coral *H. fuscescens*…”

L574: “These results may hint toward a specific attraction mechanism.”

- Did the authors mean a “specific **chemical** attraction mechanism”?

S2 Appendix Fig. 2 description: “The black bar indicate chromophore.”

- Should be: “The black bar indicate**s** **the** chromophore.”

S3 Appendix: “…observed under an **epifluorescent** microscope (BX50, Olympus, Tokyo, Japan).”

- Should be: “…observed under an **epifluorescence** microscope (BX50, Olympus, Tokyo, Japan).”
