# Supplementary material for: Can Acropora tenuis larvae attract native Symbiodiniaceae cells by green fluorescence at the initial establishment of symbiosis?
Source: PLoS One. 2021 Jun 1;16(6):e0252514. doi: 10.1371/journal.pone.0252514 (PMC8168901; doi:10.1371/journal.pone.0252514)
Supplement: Attachment — Submitted filename: PONE-D-20-25327_Reviewer_Comments - Final.docx [file pone.0252514.s011.docx]

Major general comments:

1. I like the approach of starting by determining the action spectra of a broad range of likely and unlikely potential symbionts of *A. tenuis*, and, in general, I agree with the authors' interpretation of these data. But I do see some problems with the experimental design and the analysis of the resulting data, as follows Using an upright setup of the flask makes the general motility of the different algal strains and the vertical position of the filters two very important factors; therefore I would have expected a more suitable control, such as incubating the flasks for the same time in the dark and then taking the control samples from every position of the grid. Moreover, it also seems important to include data on the fractions of motile cells at the time of the experiment for the different strains (particularly given the data in Yamashita and Koike, 2016, where strain CCMP2466 seems to have many fewer motile cells then the others). Taking the control samples from homogenous cultures also limits the interpretation, as it seems impossible to tell, for example, whether *S. tridacnidorum* is slightly attracted to light in the blue-green spectrum or if it is just avoiding the other wavelengths more. The randomization of the filter positions seems in principle a good idea, but I find it problematic that different patterns were used between the different strains, so that the data are not strictly comparable in regard to any possible positional bias. At least I would expect a thorough statistical analysis of the data, including the positions of the filters as another factor and some discussion on the experimental setup. Additional information that should be added to the materials and methods is the light intensity. Finally, it seems important to include one more wavelength between 410 and 470 nm to define the action spectrum in more detail in this seemingly relevant range.
2. The experiments and data on the fluorescence development of the larvae (and fluorescence of juveniles and adults) seem very solid, but the images need to be revisited and appropriately adjusted (see the specific comments on Figures 3, 5 and S1). Also, it might be worth for the emission spectra to use moving averages or some other kind of smoothing to help with interpretation and limit the effect of outliers (at least for the UV-A excited larvae).
3. From the images of the larvae, it appears that the orange/red fluorescence is most prominent within the epiderm while the green fluorescence seems to be stronger in the gastroderm. Can the authors comment on this?
4. For the experiment on how exposure of larvae to *S. microadriaticum* and *D. trenchii* affects the fluorescence, I would recommend providing a bit more context in the Results section and including, if possible, also data on the efficiency of the infection and algal numbers within the larvae.
5. In the current manuscript, the data on the different genes for fluorescent proteins and the protein analysis are rather hidden; I would suggest at least mentioning them in the Results section. Moreover, I wondered if it is possible to predict which of the proteins are CFP-like and which GFP-like by, e.g., structural modeling and comparing their chromophore-forming residues? This would be interesting in the context that for some RFPs (e.g., DsRed) it has been observed that they can form an intermediate structure during chromophore maturation that shows blue fluorescence (Bravaya et al. 2012, "Insight into the common mechanism of the chromophore formation in the red fluorescent proteins: the elusive blue intermediate revealed". J Am Chem Soc. 134:2807–2814.). It seems unlikely that the blue fluorescence in the larvae could be caused only by such an intermediate, given its intensity and timing, but it would be nice to see that the larvae are actually expressing a blue/cyan fluorescent protein. I also wonder whether the authors thought about looking at the expression of the different genes during larval development by qRT-PCR? It could be interesting to see whether there is a change in the expression patterns of different RFPs to cause the shift in the peak red/orange emission.
6. My biggest concern is about the final infection experiment under different light sources. First, I couldn't find information on the light intensities for the different wavelengths and how they compare to natural environmental levels. But, mainly, I find this experiment to be somewhat disconnected from the rest of the manuscript. The previous experiments suggest highest attraction of the two algal strains to blue-to-cyan fluorescence and show that larvae show a peak blue fluorescence between 450 and 470 nm under UV-A excitation, which fits nicely to the action spectra of the algae. The other peak is in the 600-625 nm range. Under violet-blue excitation, the larvae show a broad emission of 580 to 620 nm (with the peak shifting from 620 nm to 580 nm over the time course) and a relatively discrete green peak at about 520 nm, a wavelength which caused only low to moderate phototaxis of the algae in the first experiment. But now the authors chose a blue, 470-nm LED that only excites the green and red fluorescence emission of the larvae as they showed earlier. As a control, a red, 660-nm LED is used, a wavelength that is completely lacking from the initial phototaxis experiment. Surely, the experiment shows differences in the infection of larvae by the algal strains under the two light sources and might suggest other factors involved in attraction of (especially) *D. trenchii* to the larvae, but I do not understand why the authors did not perform a similar experiment under UV-A light; UV-A LEDs between 365 and 405 nm are easily available for reasonable prices. And so long as the intensity is not above what the larvae face in nature, I would not expect any UV-toxicity effects, especially given the short exposure time. Moreover, I would expect additional controls such as infection in full-spectrum light (such as that from the xenon-arc lamp, adjusted to appropriate intensity) and in the dark. Without such experiments, the manuscript seems to set the stage for the hypothesis that non-green fluorescence might help coral larvae attract Symbiodiniaceae but then fails to actually test that hypothesis. I do understand the limitations and challenges of coral spawning, which may have restricted the authors' ability to do these experiments, but then I think they need to explain more clearly the reasoning of the original experiment and set it more clearly apart from the previous ones.

Minor general comments:

1. Some other technical details appear to be missing, such as the light intensities behind the filters in the first experiment, the intensities of the LED light in the infection experiment, and how the microscopy of the larvae was done.
2. I found it at times pretty hard to follow the Results section, which mostly used the rather cryptic strain designations. I think it would be better to use the species names in the text, as the strains can be looked up in the Materials and Methods section if needed.
3. There were some odd expressions, mostly regarding the algal cultures, I tried to mark them all in the specific comments. Furthermore, I would suggest reducing the frequency of the word “Symbiodiniaceae”/”symbiodiniacean” throughout the text. I understand the specificity it provides, but it makes the text very repetitive and hard to read and is mostly unnecessary because it is clear what types of algae are involved.
4. I think that some of the parts of the Results section have very abrupt (if any) transitions and lack explanations of the reasoning behind the experiments and how they connect to each other.
5. I think the supplementary tables need better explanations or labels within them; this is especially true for the different fluorescence spectra.
6. To make interpretation easier and more accessible for readers with deficiencies in color vision, I would suggest including greyscale images in the supplementary data for all of the microscopy images

Specific comments:

L67-68: “…do not have their own symbionts…”

- This is an odd expression, maybe replace by “are apo-symbiotic” or “lack symbionts”.

L80: “…harbor specific **genus** of Symbiodiniaceae;”

- “…harbor specific **genera** of Symbiodiniaceae;”

L92: “…can attract a Symbiodiniaceae culture strain”

- I think this should be “…can attract a cultured strain of Symbiodiniaceae”

L119: “Culture strains”

- “Cultured strains” or “Symbiodiniacean cultures”

L140: “The **culture** strains…”

- “The **cultured** strains”

L140-141:

- Were the strains inoculated in IMK again or plain sea water? I would assume from the previous paragraph that it was in IMK, but I think this is important information to provide, as additional nutrients in the IMK might have an effect on the phototaxis.

L149-150: “The cell culture flask, which was randomly attached to these optical filters,…”

- I would suggest writing “The cell-culture flask, to which these optical filters were attached in a random order,…”.

L160-161: “These measurements were performed three times with the randomly re-attached filters.”

- I was wondering in what way the positions were randomized between the experiments when reading this and only later saw that the order was documented in the Supplementary Dataset S2. I would suggest referencing this dataset at this point.
- Please also see my other comments for this experiment.

182: “Fluorescent micrographs…”

- “Fluorescence micrographs”

L192-195:

- I have no experience using PMAs. Are the example images shown in Figs. 3, 5 and 7 representative for which regions were measured by the PMA, or are there more possibilities to limit the measurements to only the actual mouth region? I think half a sentence clarifying that might help in the text (or just referring to the example images).
- Also, the authors do not mention any fixation, so I assume these are living larvae; are they only kept in place by the cover slip squeezing them in?

L195: “…placing a hair between the slide glass and the cover glass.”

- Should be “…placing a hair between the glass slide and the cover glass.”

L218: “…AJIS2-C2 ( S . microadriaticum ) and CCMP2556 ( D . trenchii ) cells were added…”

- “…AJIS2-C2 (*S . microadriaticum*) **or** CCMP2556 (*D . trenchii*) cells were added…”

L220-221:

- Were the numbers of algae also counted for this analysis?

L224-226:

- Was the settlement itself done in the flow-through system (and thus, under algal exposure) or in separate containers and the fragments placed in the flow-through system only once settled? This needs clarification.

L235-237: “Five individual 6-day-old apo-symbiotic larvae were put into the 12 aforementioned 100 mL glass cups with 50 mL of 0.4-µm-filtered seawater.”

- I would suggest changing the sentence slightly: “Five individual 6-day-old aposymbiotic larvae were put into each of 12 100-mL glass cups with 50 mL of 0.4-µm-filtered seawater.”

L244-245: “Visible spectrum fluorescence, including green fluorescence, can be excited under blue LED, but not red LED”

- Using the 470-nm LED also excludes all shorter wavelength blue fluorescence. See also my general comment #2.

L245-247:

- Were the larvae assessed for acquired algae directly after the exposure to the algae in the two different light treatments? If so, maybe add a ”Subsequently,…” to the begin of that sentence; if not please provide the information on when they were assessed.

L268-269: “For the infected Symbiodiniaceae cell densities within the individual larva…”

- I suggest changing the order in this sentence and replace “infected” by “exposed” to make it more precise: “For the Symbiodiniaceae cell densities within the exposed individual larva…”

L279: “Phototaxis of Symbiodiniaceae cultures”

- I suggest “Phototaxis of cultured Symbiodiniaceae”

L282: “…observed in the culture strains…”

“…observed in cultures of strains…”

L287: “The culture strains…”

- “Cultured strains…”

L305-308: “The raw intensity data was scattered; thus, the data near the peak showed fine jaggedness. However, in Fig.4 and Fig. 6, we describe the wavelength at the highest intensity recorded.”

- I do not understand the meaning or context of the second sentence. Please also see my earlier comment on applying, e.g., moving-average smoothing to the emission spectra.

L325-327:

- I think this needs one or two sentences to transition from the time-course experiments on aposymbiotic larvae to the one with infected larvae. At this point, it is not clear to me what the authors want to learn from that latter experiment.

L358-359:

- I would suggest adding arrows to the figure to point at the alga(e?); I am only able to spot one algal cell in the bottom-row larva.

L379: “The infected CCMP2556 cell numbers significantly differed…”

- I suggest “The numbers of CCMP2556 cells acquired by larvae differed…”

L384-385: “The percentage of Symbiodiniaceae cell-infected *A . tenuis* larvae and infected cell numbers within the larvae…”

- I would change this to “The percentage of infected larvae and algal numbers within each larva…”
- The age of the larvae is included in the Materials and Methods section, but it might be good to add this information in this figure legend as well.

L424: “…and 2) Symbiodiniaceae culture strain “OTcH-1”…”

- “…and 2) cultured symbiodiniacean strain “OTcH-1”…”

L426: “However, the culture strain OTcH-1 was…”

- “However, strain OTcH-1 was…”

L465: “…showed positive phototaxis to 470 and 490 nm and 470 nm, respectively,…”

- “…showed positive phototaxis to 490 nm and 470 nm, respectively,…”

L484-485: “The percentage of *S. microadriaticum* (AJIS2-C2; type A1)-infected larvae and infected cell numbers within the larvae significantly differed between LED light sources.”

- I suggest changing the sentence to “The percentage of *S. microadriaticum* (AJIS2-C2; type A1)-infected larvae and the algal cell numbers within the larvae differed significantly between LED light sources.”

L489: “…,infected *D. trenchii* cell numbers…”

- “…,cell numbers of acquired *D. trenchii*…”

L525: “…fluorescence is not only for larvae/juvenile characteristics.”

- I would suggest changing this part to “…fluorescence is not an exclusive characteristic of larvae/juvenile.”

Fig. 2:

- y-axis label should be changed to something like “Changes in cell density in response to irradiation with different wavelengths of light” as the graphs show no rates (which would be change over time) and they don’t show changes of the cells but their numbers.

Fig. 3:

- The first two images are just black squares with labels without any background noise or other signals. Judging from the 3- and 4-day images, I suspect that the contrast was set too aggressively, resulting in the complete cut-off of all weaker signals. (Please see also the comment on Figs. 5 and S1).

Fig. 4:

- y-axis label should be simplified/changed to i.e. “Relative fluorescence intensity”. I think it is clear from the manuscript text that these measurements were done using an epifluorescen**ce** microscope.
- Also, interpretation of the figure would be easier if all y scales would be the same (maybe insets could be used to present the very small intensities from the first couple of days if they are otherwise not visible).

**Fig. 5/S1 Fig.:**

- **The first four images (in both figures) are only uniformly black squares with labels on them, there is no background noise or anything in them (not even compression artifacts). These are not microscopy images; either something went wrong during creation of the figures, or they were processed improperly! Based on the spectra from Fig. 6, I would expect to still see fluorescence signal of the larvae at least for the 3- and 4-day old ones (and background fluorescence comparable to the later time points in all of them).**

This paper provides good guidelines for processing and preparing scientific image data: M. Rossner & K. M. Yamada: What's in a picture? The temptation of image manipulation . J Cell Biol 2004; 166: 11–15. doi: https://doi.org/10.1083/jcb.200406019

Fig. 8:

- Same comment for the y-axis as for Fig. 4. I think the different y-scales are OK here, maybe just mention the different scales in the legend.

Fig. 9:

- Left y-axis label needs hyphens: “Symbiodiniaceae-cell**-**infected”. Or re-order the words.
- For the right y-axis I would suggest “Symbiodiniacean cells acquired per larvae”

Appendix Figs. 1 and 2:

- As mentioned above, I would suggest mentioning the data at least briefly in the Results section in regard to the fluorescence measurements.
