# Supplementary material for: Can Acropora tenuis larvae attract native Symbiodiniaceae cells by green fluorescence at the initial establishment of symbiosis?
Source: PLoS One. 2021 Jun 1;16(6):e0252514. doi: 10.1371/journal.pone.0252514 (PMC8168901; doi:10.1371/journal.pone.0252514)
Supplement: Attachment — Submitted filename: Response_to_Reviewers.docx [file pone.0252514.s012.docx]

Dr. Anderson B. Mayfield

Academic Editor, PLOS ONE

Please find attached our revised manuscript originally titled “Possible attraction of native symbiodiniacean species to *Acropora* *tenuis* larvae caused by non-green fluorescence factors” (PONE-D-20-25327), which has been revised based on the comments in your e-mail dated Sept. 29, 2020. We express our sincere apologies for the delay in sending you this reply.

The manuscript has been revised in accordance with the constructive and insightful comments by the reviewers. This letter contains point-by-point responses to the reviewers’ comments. Specific details of our responses to all comments are listed in the “Author responses” section, and the following is a summary of major changes in the manuscript.

+++++++++++++++++++++++++++++++++++++++++++++++++++++++++++++++++++++

**Considerable changes throughout the content**

1. The reviewers are concerned about the lack of statistical analysis for the phototaxis experiment. In the revised manuscript, we applied generalized additive models to test for relationships between the wavelength and the increase/decrease in symbiodiniacean cell numbers. Wavelength significantly affected phototaxis of cultured strains AJIS2-C2 (type A1; *Symbiodinium microadriaticum*), GTP-A6-Sy (type A2 relative; *Symbiodinium natans*), CS-161 (type A3; *Symbiodinium tridacnidorum*), CCMP1633 (clade B; *Breviolum* sp.), and CS-156 (clade F; *Fugacium kawagutii*). We also added the estimated relationship between wavelength and these cultured strains based on fitted parameter estimates ± 95% confidence intervals in new supporting information S1 Fig.

2. The reviewers are also concerned with the infection test under blue and red LED lights. In this experiment, we used blue LED light with a wavelength of 470 nm. As reviewers pointed out, this wavelength may not excite larval fluorescence. We decided to remove data on the infection rate and infected symbiodiniacean cells within larvae under blue LED to avoid confusion. However, visible fluorescence of the larvae cannot be excited by red LED light at a wavelength of 660 nm. Nevertheless, the larvae acquired CCMP2556 (type D1-4; *Durusdinium trenchii*). This result indicated that fluorescence is not the only factor of symbiodiniacean cell attraction. Thus, we have retained information on infected symbiodiniacean cells within larva under red LED.

3. We have removed information on “Fluorescence of *A. tenuis* larvae with or without Symbiodiniaceae cells,” and “Fluorescence of juvenile and adult *A. tenuis*.” to avoid difficulty in reading and because omitting these data would not affect our discussion. No significant fluorescence from juvenile corals was observed. However, reviewer#1 pointed out, our data was obtained from only three juveniles. We removed fluorescence data for juveniles to avoid generalizations. The details of fluorescence spectra of adult *A*. *tenuis* corals are now moved to supporting information (S3 Appendix). We found blue and green fluorescence from adult colonies, and we discuss adult fluorescence without generalizations.

4. Our data cannot fully explain whether native symbiodiniacean cells were attracted by “non-green fluorescence factors.” However, larval maximum green fluorescence peak did not coincide with that of the phototaxis action spectra of native *S*. *microadriaticum* cells. Furthermore, CCMP2556 (type D1-4; *Durusdinium trenchii*) cells were acquired by larvae even under red LED light. Recently, green fluorescence of corals was shown to play an important role in attracting symbionts. However, our results indicate the possibility that various factors, not limited to green fluorescence, are involved in symbiodiniacean cell attraction. Unfortunately, we could not determine the attractants within the scope of our current work. However comprehensive research that includes not only green fluorescence but also other fluorescence and other attractants (e.g., chemical compounds) is needed, to elucidate the attraction mechanisms. We have revised our manuscript to address the reviewers’ concerns and avoid unnecessary discussion. The manuscript title has been revised to “Can *Acropora tenuis* larvae attract native Symbiodiniaceae cells by green fluorescence at the initial establishment of symbiosis?”

+++++++++++++++++++++++++++++++++++++++++++++++++++++++++++++++++++++

**Author’s responses (point-by-point responses)**

**comment from Reviewer #1:**

1-1. My major concerns for this paper are that the samples sizes are insufficient for the majority of the experiments performed. Especially since it is known there is substantial genotypic and phenotypic variability with regards to coral larval fluorescence (Meyer et al 2011, Kenkel et al 2011, etc.). Therefore, the low samples sizes here (3 larvae for photographs, 3 juveniles, 3 adults, no replicates for the infection experiment) does not lend me to trust these results are representative of phenotypes in this system.

Our response:

Meyer et al., 2011 (Mol. Ecol.20:3599-3616) demonstrated that different larval families display extensive variation in gene expression depending on their genetic background, including qualitative differences. Furthermore, Kenkel et al. (2011) (Proc. R. Soc. B. 278:2691-2697) demonstrated that 18% of larval color variation was explained by parental effects. These results show that larval gene expression and the resulting fluorescence color can differ among parental combinations. In our study, three larvae were sacrificed and observed every observation day. Thus, a total of 36 larvae were observed (Figs. 4, 6). These larvae were obtained from nine parental *A. tenuis* colonies. Thus, it is plausible that the observed larvae had mixed characteristics from nine parental characteristics. As the reviewer pointed out, larval fluorescence patterns can differ. A good example is larval orange fluorescence. We occasionally found faint orange fluorescence larvae; however, even these larvae have green and broad blue fluorescence. Larvae with faint orange fluorescence are recognized as “greenish” larvae under fluorescence microscopy. Although the intensity can differ among observed larvae, the peak wavelength of broad blue and green fluorescence was well congruent. Thus, fluorescence proteins are present, even if their expression levels vary. Please see our new S2 Appendix file. Fluorescence data from juveniles and adults were observed from only three colonies in our study. We decided to remove the results of juveniles in the revised manuscript. Data from adult colonies has been moved to supporting information. Furthermore, our infection test was conducted only once with triplicate glass cups. The data indicated that larvae can acquire CCMP2556 (type D1-4; *Durusdinium trenchii*) cells even under red LED light. This cultured strain was intrinsically attracted to and acquired by apo-symbiotic *A*. *tenuis* larvae (Yamashita et al., 2014 *PLOS ONE* <https://doi.org/10.1371/journal.pone.0097003>). However, we could not find specific phototaxis patterns of this strain. These results may indicate the attraction mechanisms other than green fluorescence. In the revised manuscript, we have cited the studies by Meyer et al. (2011) and Kenkel et al. (2011).

1-2. Further, there was not sufficient replication for the symbiodiniacean phototaxis experiments (were different cohorts of symbiodinaceans examined? on different days?) as well and some clarity on that is warranted. The biggest concern is the lack of any statistical test on the phototaxis experiments (Figure 2) from what I can tell. Further, the authors deduce positive or negative phototaxis of certain types (Durisdinium for example) where I worry a statistical test would invalidate that claim considering the breadth of the error bars. While the small samples sizes are unfeasible to be changed at this conjecture, I think at least discussion or consideration of this is warranted.

Our response:

We used eight symbiodiniacean cultured strains. These strains were used in previous infection experiments with *A. tenuis* larvae (Yamashita et al., 2014 *PLOS ONE*, <https://doi.org/10.1371/journal.pone.0097003>); thus, we already know the strains that can be acquired by *A. tenuis* larvae. Using these strains, we think that the effect of symbiodiniacean cell attraction by apo-symbiotic larvae can be clearly demonstrated. In the revised manuscript, we applied generalized additive models to determine the effect of wavelength on phototaxis. Please see Response 1 in “Considerable changes throughout the content” in the first page. As reviewer pointed out, wavelength did not significantly affect phototaxis of *Durusdinium trenchii*.

1-3. Line 115-116: It would be good to review the recommendations for this journal, but typically the permit information would go at the end. It is a distraction for the reader where it is now.

Our response:

Thank you for your suggestion. We have moved this information to the appropriate section.

1-4. Line 159: Were these three repeated measurements with different cohorts of Symbiodinaceae or the same exact sample of Symbiodinaceae? I worry there is not enough replication for this study.

Our response:

Please see our response to comment 1-2. We examined symbiodiniacean cell phototaxis three times with randomly re-attached filters. Eight cultured Symbiodiniaceae strains were used for this observation. Each strain was a clone culture. Thus, if each strain were divided into three bottles, it is not so effective as replicate. Furthermore, we think that comparisons of phototaxis among strains are unnecessary. We focused on whether the cultured strains, especially native symbionts, demonstrate positive phototaxis to green light. We applied GAM to test for the effect of wavelength on phototaxis of each culture strain separately.

1-5. Figure 1: I don’t understand how samples could have been taken from each grid if the culture flask was standing upright, as it appears in Figure 1. If you then put the flask on it’s side to take a sample of each grid wouldn’t that redistribute the symbionts within the flask?

Our response:

During observation, the culture flasks were always in an upright position (Fig. 1c). After turning on the light for 10 min, the cap of the flask was carefully unscrewed. Then, a Pasteur pipette was carefully inserted from flask’s mouth. By observing the pipette position from the side (namely from the opposite side of the filter-attached side, Fig. 1d), the tip of the pipette was carefully moved to near the center of the grid attached to each optical filter. This procedure was not difficult.

1-6. Figure 2. Where are the statistics reported to tests significant deviations from null expectations?

Our response:

In the original manuscript, we simply defined positive phototaxis as more than the 100% line. In the revised manuscript, we applied GAM to test for the effect of wavelength on the increase/decrease in each symbiodiniacean cell numbers. Please see Response 1 in “Considerable changes throughout the content.”

1-7. Figure 4: you cannot distinguish the colors denoting differences between larva 1, 2, and 3. The figure needs to be replotted to clarify that. In addition, the resolution appears to be too low. Further, there appears to be substantial variation in fluorescence intensity between the three larvae measured. and many times the larvae is the exact same as the background (from what I can tell). This leads me to believe there was not sufficient sample sizes to truly deduce the larval fluorescent signal from the background as well as represent the existing variation among larval cohorts.

Our response:

We apologize for this. The resolutions of the figures were automatically reduced by the submission system for review. We have attached revised Fig. 4. To observe larval fluorescence, we sacrificed three individuals each day, and the total number of observed larvae was 36 individuals. All intensity data are from completely different individuals. The intensity varied among individuals; however, the peaks of green, broad orange, and broad blue fluorescence were consistent among the observed larvae. Although we observed only three larvae per observation day, this result indicates that the larval fluorescence patterns were accurately measured. Please also see Response 1-1 in the Author’s responses (point-by-point responses) section. The fluorescent intensity of the “background” was almost 0, and these data are plotted on the x-axis.

revised Fig 4

********************************************************************************************

Reviewer #2:

Major general comments:

2-1. Using an upright setup of the flask makes the general motility of the different algal strains and the vertical position of the filters two very important factors; therefore I would have expected a more suitable control, such as incubating the flasks for the same time in the dark and then taking the control samples from every position of the grid. Moreover, it also seems important to include data on the fractions of motile cells at the time of the experiment for the different strains (particularly given the data in Yamashita and Koike, 2016, where strain CCMP2466 seems to have many fewer motile cells then the others). Taking the control samples from homogenous cultures also limits the interpretation, as it seems impossible to tell, for example, whether S. tridacnidorum is slightly attracted to light in the blue-green spectrum or if it is just avoiding the other wavelengths more. The randomization of the filter positions seems in principle a good idea, but I find it problematic that different patterns were used between the different strains, so that the data are not strictly comparable in regard to any possible positional bias. At least I would expect a thorough statistical analysis of the data, including the positions of the filters as another factor and some discussion on the experimental setup. Additional information that should be added to the materials and methods is the light intensity. Finally, it seems important to include one more wavelength between 410 and 470 nm to define the action spectrum in more detail in this seemingly relevant range.

Our response:

We examined symbiodiniacean cell phototaxis three times with randomly re-attached filters. In our observation, bias in cell numbers either up or down the side of the culture flask was not observed. For example, in strain AJIS2-C2 (type A1; *Symbiodinium* *microadriaticum*), no significant positive phototaxis was observed to light at a wavelength of 530 nm. This optical filter was located on the first line at the 1st observation, second line at the 2nd observation, and third line at the 3rd observation. The mean cell numbers at the 1st, 2nd, and 3rd observations were 37.7, 33, and 30 cells, respectively. These results indicated that the position of the optical filters and cell numbers were independent. As reviewer pointed out, motile cell numbers can differ among cultured strains. Thus, we showed phototaxis as a “percentage” calculated as follows:

(average cell numbers of after lamp irradiation) / (average initial cell numbers) × 100.

Furthermore, we think that the comparison of phototaxis levels among the cultures is unnecessary. In the present study, we focused on whether the cultured strains, especially native symbionts, possess positive phototaxis to green light. Differences of motile cell numbers are not so important because the discussion focuses on single strains. Regarding the statistics, please see Response 1 in “Considerable changes throughout the content” in the first page.

We appreciate this comment from reviewer. To determine the symbiodiniacean phototaxis pattern to full wavelength light, optical filters between 410 and 470 nm (and also long wavelength part) are needed. However, we focused on whether the culture strains, especially native symbionts, possess positive phototaxis to green light. Thus, we used filter sets of 330–385 nm, 400–410 nm, 470 nm, 490 nm, 510 nm, 530 nm, 550 nm, and 570 nm. Unfortunately, data on light intensity was not available. Aihara et al. (2019) (*PNAS*, 116: 2118–2123) demonstrated that the Symbiodiniaceae cultured strain OTcH-1 showed positive phototaxis to 400–510-nm weak light, whereas this culture strain showed negative phototaxis to 375–475-nm strong light. In the present study, there was no significant negative phototaxis at 375–475 nm. Thus, no negative effects were expected from strong light intensity. In a future study, to determine the Symbiodiniaceae eco-physiology, we will observe Symbiodiniaceae phototaxis under full wavelength light and various intensities.

2-2. The experiments and data on the fluorescence development of the larvae (and fluorescence of juveniles and adults) seem very solid, but the images need to be revisited and appropriately adjusted (see the specific comments on Figures 3, 5 and S1). Also, it might be worth for the emission spectra to use moving averages or some other kind of smoothing to help with interpretation and limit the effect of outliers (at least for the UV-A excited larvae).

Our response:

Thank you for your suggestion. The PMA data are scattered data. We agree with your suggestion that smoothing should be used. However, in the smoothing process, numerous data will be lost, and we are concerned about image manipulation. Furthermore, in Fig. 3 and 5 (also newS3 Fig), as pointed out by the reviewer, 1-, 2-, and 3 day-old larvae could not be visibly recognized. All figures were taken under the same exposure time and without any digital processing (brightness was adjusted in Fig. 5). This is because we want to show visible changes in the trend of larval fluorescence. Thus, we spent 2 years trying to find the optimal exposure time, that is, fluorescence in 21-day-old larvae does not cause saturation. However, this exposure time is short, and weak fluorescence if 1–3-day-old larvae do not appear in the image. We have added this information in the figure legends. The below image shows the same position of 1-day-old coral in Figs. 3 and 5 taken with a longer exposure time.

blue violet excitation	UV-A excitation

2-3. From the images of the larvae, it appears that the orange/red fluorescence is most prominent within the epiderm while the green fluorescence seems to be stronger in the gastroderm. Can the authors comment on this?

Our response:

We agree with this observation. However, to determine the fluorescence localization pattern, three-dimensional observations are necessary.

2-4. For the experiment on how exposure of larvae to S. microadriaticum and D. trenchii affects the fluorescence, I would recommend providing a bit more context in the Results section and including, if possible, also data on the efficiency of the infection and algal numbers within the larvae.

Our response:

Please see Response 2 in “Considerable changes throughout the content” in page 1.

2-5. In the current manuscript, the data on the different genes for fluorescent proteins and the protein analysis are rather hidden; I would suggest at least mentioning them in the Results section. Moreover, I wondered if it is possible to predict which of the proteins are CFP-like and which GFP-like by, e.g., structural modeling and comparing their chromophore-forming residues? This would be interesting in the context that for some RFPs (e.g., DsRed) it has been observed that they can form an intermediate structure during chromophore maturation that shows blue fluorescence (Bravaya et al. 2012, "Insight into the common mechanism of the chromophore formation in the red fluorescent proteins: the elusive blue intermediate revealed". J Am Chem Soc. 134:2807–2814.). It seems unlikely that the blue fluorescence in the larvae could be caused only by such an intermediate, given its intensity and timing, but it would be nice to see that the larvae are actually expressing a blue/cyan fluorescent protein. I also wonder whether the authors thought about looking at the expression of the different genes during larval development by qRT-PCR? It could be interesting to see whether there is a change in the expression patterns of different RFPs to cause the shift in the peak red/orange emission.

Our response:

Thank you for your comment. In the revised manuscript, we included data on the different genes encoding fluorescent proteins and protein analysis in the Results section. We think that determination of the actual color of expressed fluorescent proteins is the next step. A study on the differences of larval gene expression between with and without Symbiodiniaceae symbionts as well as age has been accepted in *Genome Biology and Evolution* (Yoshioka et al., 2020, <https://doi.org/10.1093/gbe/evaa240>). In this analysis, we could not find differences of expression in genes encoding fluorescent proteins. However, we selected genes with considerable differences in their expression. An analysis focusing on specific genes is currently underway.

2-6. My biggest concern is about the final infection experiment under different light sources. First, I couldn't find information on the light intensities for the different wavelengths and how they compare to natural environmental levels. But, mainly, I find this experiment to be somewhat disconnected from the rest of the manuscript. The previous experiments suggest highest attraction of the two algal strains to blue-to-cyan fluorescence and show that larvae show a peak blue fluorescence between 450 and 470 nm under UV-A excitation, which fits nicely to the action spectra of the algae. The other peak is in the 600-625 nm range. Under violet-blue excitation, the larvae show a broad emission of 580 to 620 nm (with the peak shifting from 620 nm to 580 nm over the time course) and a relatively discrete green peak at about 520 nm, a wavelength which caused only low to moderate phototaxis of the algae in the first experiment. But now the authors chose a blue, 470-nm LED that only excites the green and red fluorescence emission of the larvae as they showed earlier. As a control, a red, 660-nm LED is used, a wavelength that is completely lacking from the initial phototaxis experiment. Surely, the experiment shows differences in the infection of larvae by the algal strains under the two light sources and might suggest other factors involved in attraction of (especially) D. trenchii to the larvae, but I do not understand why the authors did not perform a similar experiment under UV-A light; UV-A LEDs between 365 and 405 nm are easily available for reasonable prices. And so long as the intensity is not above what the larvae face in nature, I would not expect any UV-toxicity effects, especially given the short exposure time. Moreover, I would expect additional controls such as infection in full-spectrum light (such as that from the xenon-arc lamp, adjusted to appropriate intensity) and in the dark. Without such experiments, the manuscript seems to set the stage for the hypothesis that non-green fluorescence might help coral larvae attract Symbiodiniaceae but then fails to actually test that hypothesis. I do understand the limitations and challenges of coral spawning, which may have restricted the authors' ability to do these experiments, but then I think they need to explain more clearly the reasoning of the original experiment and set it more clearly apart from the previous ones.

Our response:

Thank you for your comment. Please see Responses 2 and 4 in “Considerable changes throughout the content” in page 1.

Minor general comments:

2-7. Some other technical details appear to be missing, such as the light intensities behind the filters in the first experiment, the intensities of the LED light in the infection experiment, and how the microscopy of the larvae was done.

Our response:

Please see our response for comment 2-1. The microscopic observation was conducted according to our previous study (Yamashita et al., 2014, *PLOS ONE*, <https://doi.org/10.1371/journal.pone.0097003>), i.e., all larvae within each experimental cup were observed in the presence of symbiodiniacean cells using an epifluorescent microscope (BX50, Olympus, Tokyo, Japan; 400–410 nm ex.). Individual larvae were gently pressed between a glass slide and cover slip, and the autofluorescence of symbiodiniacean cell chlorophyll a within larval bodies was observed. We have included this information in the revised manuscript.

2-8. I found it at times pretty hard to follow the Results section, which mostly used the rather cryptic strain designations. I think it would be better to use the species names in the text, as the strains can be looked up in the Materials and Methods section if needed.

Our response:

We have updated the text accordingly.

2-9. There were some odd expressions, mostly regarding the algal cultures, I tried to mark them all in the specific comments. Furthermore, I would suggest reducing the frequency of the word “Symbiodiniaceae”/”symbiodiniacean” throughout the text. I understand the specificity it provides, but it makes the text very repetitive and hard to read and is mostly unnecessary because it is clear what types of algae are involved.

Our response:

We have updated the text accordingly.

2-10. I think that some of the parts of the Results section have very abrupt (if any) transitions and lack explanations of the reasoning behind the experiments and how they connect to each other.

Our response:

Please see Response 3 in “Considerable changes throughout the content.”

2-11. I think the supplementary tables need better explanations or labels within them; this is especially true for the different fluorescence spectra.

Our response:

Thank you for your suggestion; we have incorporated the suggestions.

2-12. To make interpretation easier and more accessible for readers with deficiencies in color vision, I would suggest including greyscale images in the supplementary data for all of the microscopy images

Our response:

Thank you for your suggestion; we have revised the images accordingly.

2-13. L140-141: Were the strains inoculated in IMK again or plain sea water? I would assume from the previous paragraph that it was in IMK, but I think this is important information to provide, as additional nutrients in the IMK might have an effect on the phototaxis.

Our response:

We observed phototaxis in IMK medium. This observation was conducted in the same cell culture flask used for pre-incubation. We observed symbiodiniacean cells during the early-to-middle logarithmic growth stage. Thus, a sufficient amount of nutrients was present in the cell culture flask. We have updated this information.

2-14. L192-195: I have no experience using PMAs. Are the example images shown in Figs. 3, 5 and 7 representative for which regions were measured by the PMA, or are there more possibilities to limit the measurements to only the actual mouth region? I think half a sentence clarifying that might help in the text (or just referring to the example images). Also, the authors do not mention any fixation, so I assume these are living larvae; are they only kept in place by the cover slip squeezing them in?

Our response:

The measurement area of PMA was small. Fluorescence spectra were measured around the middle of each image in Fig. 3. We have included this information in the revised manuscript. Larval fluorescence was measured without any fixation.

2-15. L220-221: Were the numbers of algae also counted for this analysis?

Our response:

We needed to compress the larvae more than when measuring fluorescence using PMA to count the symbiodiniacean cell numbers within the larvae. Thus, symbiont cell numbers were not counted in these larvae. Other larvae in the same experimental cups acquired approximately 10 symbiont cells. However, this result is not of great importance, and we decided to remove this information from the revised manuscript.

2-16. L224-226: Was the settlement itself done in the flow-through system (and thus, under algal exposure) or in separate containers and the fragments placed in the flow-through system only once settled? This needs clarification.

Our response:

The larval settlement was induced in a tank without flow for 2 days. After settlement, the fragments were placed in the flow-through system. However, this result is not so important, and we decided to remove this from the revised manuscript.

2-17. L305-308: “The raw intensity data was scattered; thus, the data near the peak showed fine jaggedness. However, in Fig.4 and Fig. 6, we describe the wavelength at the highest intensity recorded.” I do not understand the meaning or context of the second sentence. Please also see my earlier comment on applying, e.g., moving-average smoothing to the emission spectra.

Our response:

Please see our response to comment 2-2.

2-18. L325-327: I think this needs one or two sentences to transition from the time-course experiments on aposymbiotic larvae to the one with infected larvae. At this point, it is not clear to me what the authors want to learn from that latter experiment.

Our response:

Thank you for your comment. We have removed these sentences from the revised manuscript.

2-19. L358-359: I would suggest adding arrows to the figure to point at the alga(e?); I am only able to spot one algal cell in the bottom-row larva.

Our response:

Please see our response to comment 2-15.

2-20. Fig. 2: y-axis label should be changed to something like “Changes in cell density in response to irradiation with different wavelengths of light” as the graphs show no rates (which would be change over time) and they don’t show changes of the cells but their numbers.

Our response:

Thank you for your comments. This result was calculated using the following formula:

(average cell number after lamp irradiation) / (average initial cell numbers) × 100

This represents the increase/decrease in the cell numbers near the grids attached to each wavelength band-pass filter.

2-21. Fig. 3: The first two images are just black squares with labels without any background noise or other signals. Judging from the 3- and 4-day images, I suspect that the contrast was set too aggressively, resulting in the complete cut-off of all weaker signals. (Please see also the comment on Figs. 5 and S1).

Our response:

Please see our response to comment 2-2.

2-22. Fig. 4: y-axis label should be simplified/changed to i.e. “Relative fluorescence intensity”. I think it is clear from the manuscript text that these measurements were done using an epifluorescence microscope. Also, interpretation of the figure would be easier if all y scales would be the same (maybe insets could be used to present the very small intensities from the first couple of days if they are otherwise not visible).

Our response:

Thank you for your comment. We have changed the label of the y-axis. One- and 2-day-old larvae have very little fluorescence, and the fluorescence intensity was almost the same as the background intensity. We have added the sentence “The y-axis values of the 16- and 21-day old larvae are different from the others” in the revised figure legend.

2-23. Fig. 5/S1 Fig.: The first four images (in both figures) are only uniformly black squares with labels on them, there is no background noise or anything in them (not even compression artifacts). These are not microscopy images; either something went wrong during creation of the figures, or they were processed improperly! Based on the spectra from Fig. 6, I would expect to still see fluorescence signal of the larvae at least for the 3- and 4-day old ones (and background fluorescence comparable to the later time points in all of them).

This paper provides good guidelines for processing and preparing scientific image data: M. Rossner & K. M. Yamada: What's in a picture? The temptation of image manipulation . J Cell Biol 2004; 166: 11–15. doi: <https://doi.org/10.1083/jcb.200406019>

Our response:

Please see our response to comment 2-2.

All of the suggestions below have been incorporated into the revised manuscript as applicable.

Specific comments:

L67-68: “…do not have their own symbionts…”

-	This is an odd expression, maybe replace by “are apo-symbiotic” or “lack symbionts”.

L80: “…harbor specific genus of Symbiodiniaceae;”

-	“…harbor specific genera of Symbiodiniaceae;”

L92: “…can attract a Symbiodiniaceae culture strain”

-	I think this should be “…can attract a cultured strain of Symbiodiniaceae”

L119: “Culture strains”

-	“Cultured strains” or “Symbiodiniacean cultures”

L140: “The culture strains…”

-	“The cultured strains”

L149-150: “The cell culture flask, which was randomly attached to these optical filters,…”

-	I would suggest writing “The cell-culture flask, to which these optical filters were attached in a random order,…”.

L160-161: “These measurements were performed three times with the randomly re-attached filters.”

-	I was wondering in what way the positions were randomized between the experiments when reading this and only later saw that the order was documented in the Supplementary Dataset S2. I would suggest referencing this dataset at this point.

-	Please also see my other comments for this experiment.

182: “Fluorescent micrographs…”

-	“Fluorescence micrographs”

L195: “…placing a hair between the slide glass and the cover glass.”

-	Should be “…placing a hair between the glass slide and the cover glass.”

L218: “…AJIS2-C2 ( S . microadriaticum ) and CCMP2556 ( D . trenchii ) cells were added…”

-	“…AJIS2-C2 (S . microadriaticum) or CCMP2556 (D . trenchii) cells were added…”

L235-237: “Five individual 6-day-old apo-symbiotic larvae were put into the 12 aforementioned 100 mL glass cups with 50 mL of 0.4-µm-filtered seawater.”

-	I would suggest changing the sentence slightly: “Five individual 6-day-old aposymbiotic larvae were put into each of 12 100-mL glass cups with 50 mL of 0.4-µm-filtered seawater.”

L244-245: “Visible spectrum fluorescence, including green fluorescence, can be excited under blue LED, but not red LED”

-	Using the 470-nm LED also excludes all shorter wavelength blue fluorescence. See also my general comment #2.

L245-247:

-	Were the larvae assessed for acquired algae directly after the exposure to the algae in the two different light treatments? If so, maybe add a ”Subsequently,…” to the begin of that sentence; if not please provide the information on when they were assessed.

L268-269: “For the infected Symbiodiniaceae cell densities within the individual larva…”

-	I suggest changing the order in this sentence and replace “infected” by “exposed” to make it more precise: “For the Symbiodiniaceae cell densities within the exposed individual larva…”

L279: “Phototaxis of Symbiodiniaceae cultures”

-	I suggest “Phototaxis of cultured Symbiodiniaceae”

L282: “…observed in the culture strains…”

“…observed in cultures of strains…”

L287: “The culture strains…”

-	“Cultured strains…”

L379: “The infected CCMP2556 cell numbers significantly differed…”

-	I suggest “The numbers of CCMP2556 cells acquired by larvae differed…”

L384-385: “The percentage of Symbiodiniaceae cell-infected A . tenuis larvae and infected cell numbers within the larvae…”

-	I would change this to “The percentage of infected larvae and algal numbers within each larva…”

-	The age of the larvae is included in the Materials and Methods section, but it might be good to add this information in this figure legend as well.

L424: “…and 2) Symbiodiniaceae culture strain “OTcH-1”…”

-	“…and 2) cultured symbiodiniacean strain “OTcH-1”…”

L426: “However, the culture strain OTcH-1 was…”

-	“However, strain OTcH-1 was…”

L465: “…showed positive phototaxis to 470 and 490 nm and 470 nm, respectively,…”

-	“…showed positive phototaxis to 490 nm and 470 nm, respectively,…”

L484-485: “The percentage of S. microadriaticum (AJIS2-C2; type A1)-infected larvae and infected cell numbers within the larvae significantly differed between LED light sources.”

-	I suggest changing the sentence to “The percentage of S. microadriaticum (AJIS2-C2; type A1)-infected larvae and the algal cell numbers within the larvae differed significantly between LED light sources.”

L489: “…,infected D. trenchii cell numbers…”

-	“…,cell numbers of acquired D. trenchii…”

L525: “…fluorescence is not only for larvae/juvenile characteristics.”

-	I would suggest changing this part to “…fluorescence is not an exclusive characteristic of larvae/juvenile.”

Fig. 8:

-	Same comment for the y-axis as for Fig. 4. I think the different y-scales are OK here, maybe just mention the different scales in the legend.

Fig. 9:

-	Left y-axis label needs hyphens: “Symbiodiniaceae-cell-infected”. Or re-order the words.

-	For the right y-axis I would suggest “Symbiodiniacean cells acquired per larvae”

Appendix Figs. 1 and 2:

-	As mentioned above, I would suggest mentioning the data at least briefly in the Results section in regard to the fluorescence measurements.
