# Supplementary material for: Can Acropora tenuis larvae attract native Symbiodiniaceae cells by green fluorescence at the initial establishment of symbiosis?
Source: PLoS One. 2021 Jun 1;16(6):e0252514. doi: 10.1371/journal.pone.0252514 (PMC8168901; doi:10.1371/journal.pone.0252514)
Supplement: Attachment — Submitted filename: Response_to_Reviewers_2nd.docx [file pone.0252514.s014.docx]

The manuscript has been revised in accordance with the constructive and insightful comments provided by the reviewers. This letter contains point-by-point responses to the reviewers’ comments.

According to the comment 2-3 from Reviewer #2, we have replaced some micrographs in the Fig 3 and Fig 5, and also S2 Fig. Furthermore, we re-checked the all data, and we found a mistake in the statistical value on the analysis result for the intensity of larval green fluorescence. Thus, we have corrected this value (Δdeviance = 50.77, Δdf = 1, p = 1.039 × 10^−12^) to (Δdeviance = 40.58, Δdf = 1, p = 1.885 × 10^−10^) (Line 327-328 in the revised manuscript). However, these modifications do not affect the discussion.

The revised manuscript was edited for English language, grammar, punctuation, and spelling by Enago, the editing division of Crimson Interactive Pvt. Ltd.

**Authors’ responses (point-by-point)**

**Comment from Reviewer #1:**

1-1. I think there should be more of a discussion of the known chemical cues involved in Symbiodiniaceae attraction to corals in the introduction. And on that note, since the results are clear that there are other factors besides fluorescence involved, integrating a discussion of these chemical signals is warranted. Finally, it is interesting that there is no phototaxis of Durisdinium, despite it’s high infectivity (not necessarily substantiated by the results here, but in other papers (Abrego, Van Open and Willis Molecular Ecology 2009, as one example). Therefore, it’s possible that different symbionts have vastly different mechanisms of detecting corals and modes of infectivity. I think the paper could be improved by discussion of these results in this broader context.

**Our response:**

In the revised manuscript, we have included the observational results to chemical cues from the study by Pasternak et al.; we have added this to the Introduction and Discussion sections. Although *Durusdinium* symbionts are usually found in *Acropora* juveniles, phototaxis was not observed in this alga in the present study. This result suggests the possibility of chemical cues. However, the effectiveness of the attractant (chemical compounds and also fluorescence) is still unclear in natural environments. In the revised manuscript, we have added some discussion without generalizations.

Line 84-88

These properties may assist encounters between symbionts and apo-symbiotic coral larvae or juveniles. For example, Pasternak et al. [19] demonstrated that symbiodiniacean cells are attracted to exudate-solution drops of apo-symbiotic juvenile polyps of soft coral *Heteroxenia fscescens*.

Line 501-505

In the present study, clear positive phototaxis to specific wavelength light was not found for the *D*. *trenchii* cultured strain. However, genus *Durusdinium* including *D*. *trenchii* were often found within the *Acropora* larvae/juveniles [10,13,14,46,47]. These results indicate that not only larval fluorescence, but also other factors are involved in the attraction of *D*. *trenchii* cells.

Line 527-537

Thus, various factors, including spacious distances and the effect of water flow, should be taken into account between or around apo-symbiotic corals and their native symbionts in the environments. A chemotaxis experiment using soft coral *H*. *fuscescens* polyps and their symbionts demonstrated that the symbiont cells swam toward the polyp against flow velocities of up to 0.5 mm s^−1^; however, the cells must be within a distance in the order of a centimeter to decimeter from the polyp to sense the chemoattractant due to the dispersion of the substance [49]. In fluorescent attraction, Aihara et al. [20] demonstrated that 8-mm^2^ *E*. *aspera* coral fragments can attract symbiodiniacean cells within a 95-mm diameter plastic dish. However, it is still unclear how far *A*. *tenuis* larvae/juveniles can attract native symbiont species using chemical and/or fluorescent attractants in the natural environments.

Line 572-576

Other factors could also be considered. For example, fluorescence variation could be associated with preferential uptake of Symbiodiniaceae types [35]. In the case of soft coral, motile symbiont cells are attracted to solutions containing exudates of apo-symbiotic juvenile polyps but not to those of adult polyps [19]. These results may hint toward a specific attraction mechanism.

1-2. Line 58-59: I would recommend stating the conclusions that while there is some evidence of phototaxis to light in S. microadriatricum, this phototaxis does not appear to play a role in attraction to coral larvae (at least of this species of coral). Your results also found that the high infectivity of Durisdinium was not explained by phototaxis but is likely driven by another mechanism.

**Our response:**

We have added the following statement to the revised manuscript Line 52-53: “The green fluorescence of *A. tenuis* larvae may not play a role in initial attraction of native symbionts.”

1-3. Line 215: Unclear here how many colonies contributed to the genetic diversity within this new pool of larvae.

**Our response:**

We apologize for this. We used 10 mature *A*. *tenuis* colonies. We have included this in the revised manuscript Line 223: “we prepared another batch of *A*. *tenuis* larvae following Suzuki [25]. Ten parental colonies were collected from Sekisei lagoon,///”

1-4.Lines 356-358: These infection numbers are extremely low so not likely to be particularly relevant ecologically, especially since larvae can simply be eating the cells rather than taking them up into their cells. Because the numbers are so low I don’t really believe the symbionts are being acquired. I think discussion of the caveats of these results is warranted or they should be taken out.

**Our response:**

In the present study and also in our previous study (Yamashita et al., 2014, PLOS ONE, <https://doi.org/10.1371/journal.pone.0097003>), the infection experiments were conducted under low symbiodiniacean cell density conditions that were almost identical to the natural environments; this is because the larvae often accidentally acquire “free-living specialist” Symbiodiniaceae cells if the supplied cells are abundant, but they do not acquire these cells under low cell density conditions (140–1400 cells/L; Yamashita et al., 2014). Conversely, larvae can acquire “native” symbionts even under low cell density conditions (140–­1400 cells/L), and the number of acquired native symbiont cells within larvae does not drastically change between low cell density conditions (140–1400 cells/L) and high cell density conditions (14000 cells/L; Yamashita et al., 2014). These results indicate that the symbiont acquisition process is not a simple Symbiodiniaceae cell density-dependent larval predatory activity. As the reviewer noted, it is unclear whether acquired symbiont cells within larvae are eaten or not; however, as the first step to establish symbiosis, the larvae need to acquire native symbiodiniacean cells into their body. In the case of native symbionts, once the algal cells are acquired by the larvae, they proliferate within the larval body and establish symbiosis (Yamashita et al., 2018, <https://doi.org/10.3354/meps12474> ). The environmental symbiodiniacean cell densities are usually low (Yamashita et al., 2013, <https://doi.org/10.1007/s00338-012-0980-2>); thus, we believe it would be difficult for larvae to acquire hundreds or thousands of symbiont cells in a few hours.

1-5. Line 514-516: Does this refer to a specific study or your own results? I don’t believe the infection results presented in this study are enough to warrant this statement.

**Our response:**

We apologize for this. We have included the reference in the revised manuscript Line 568-569; “However, apo-symbiotic *A*. *tenuis* larvae certainly attracted native symbionts in proximity of laboratory experimental levels [14].”

All of the below-mentioned suggestions have been incorporated into the revised manuscript as applicable.

Line 39: remove comma after cells.

Line 55: Confused here, since you are specifying using red LED light and you did not test for green light. If I understand you correctly, I think you should remove “including green fluorescence” from this statement.

Line 350: This sentence should be adjusted for clarity. For example, “We observed 15 A. tennis individuals supplied with either S. microadriatricum or D. trenchii.”

Line 409: change to “comparison”.

＊＊＊＊＊＊＊＊＊＊＊＊＊＊＊＊＊＊＊＊＊＊＊＊＊＊＊＊＊＊＊＊＊＊＊＊＊＊＊

**Comment from Reviewer #2:**

Major general comments:

2-1. I still would like to see measured light intensities for the first experiment, in the simplest set up just the different filters between the Xenon lamp and the sensor.

**Our response:**

In the present study, we focused on whether the cultured strains, especially native symbionts, possess positive phototaxis to green light. Unfortunately, data on light intensity was not available. In a future study, to determine Symbiodiniaceae eco-physiology, we will observe Symbiodiniaceae phototaxis under full wavelength light and various intensities. In the revised manuscript, we have added that our observations are still insufficient to clarify the relationship between phototaxis and initial symbiosis establishment.

Line 521-524

Nonetheless, the current phototaxis analysis was conducted using limited wavelengths because we focused on whether native symbionts possess positive phototaxis to green light. Thus, further analysis is required to conclude on the above-proposed mechanisms.

2-2. For presenting the data on the phototaxis spectra of the different algal strains, I would recommend indicating the strains with statistically significant responses in the figure. Is there any way to obtain wavelength-specific significances of the responses from the analysis? That would make the graphical representation easy (asterisk above the bars) and also give another layer of useful information as the paper aims at untangling the responses to the different light qualities. Additionally, I would suggest including the details from S1 Fig in the results paragraph to point to the different wavelength responses, the easiest way to do that might be by just giving the wavelength ranges with a positive and a negative response. Also, I would recommend using the same y-axis scale for all graphs in S1 Fig. to make it more easy to compare them visually.

**Our response:**

Thank you for your suggestion. In the revised Fig 2, we have marked the cultured strains that showed a significant relationship between light wavelength and phototaxis. In the GAM analysis, although the peak of phototaxis can be predicted, it is difficult to compare the strength of phototaxis among the wavelengths. In the revised manuscript, we have added the estimated peak phototaxis action wavelength (Line 286-292 in the revised manuscript). We agree with your suggestion to use the same y-axis scale in S1 Fig when comparing phototaxis among the tested strains. However, as addressed in the first round of reviews, in the present study, phototaxis comparisons across strains are not assumed. Since the cell numbers differed across the tested strains, using the same y-axis scale may be misleading. In our manuscript, the discussion focuses on single strains. And the S1 Fig indicates the estimated phototaxis pattern of each tested cultured strain.

2-3. My concern about the fluorescence images of the 1 to 4-day old larvae (Fig. 3 and Fig. 5) was not about the absence of larval fluorescence, it was mainly about the absence of any (!) background noise (compare the background for the older larvae vs. that of any of the first four days) and the absence of finer graduations in the 3 and 4-day images under violet-blue excitation where the intensity between areas which show fluorescence drops directly to 0 (see the included line scan from 4 vs. 5 days). This indicates to me that the histogram of the raw images was somehow trimmed (by adjusting brightness & contrast or applying background subtraction etc.) before converting it to the final images. This does not change the validity of the results and is merely a technical issue. But I think it is important to follow good practices in image processing and presentation and things like this can easily happen by some hidden default settings in image processing software (e.g., after re-installation of the software), especially when converting from 16-bit to 8-bit or RGB images.

**Our response:**

We sincerely appreciate this comment. We have rechecked the original figure files used for Fig 3, 5, and S3 Fig, and found the signs for digital black balance in the micrographs of 1 to 4­-day-old larvae. We have changed the micrograph for 4-day-old larvae with black balance to that without black balance. The micrographs of 1 to 3-day-old larvae have been changed to micrographs taken under 500 ms exposure time with digital black balance because the fluorescence intensity of these larvae was extremely low.

2-4. I strongly oppose the removal of the infection results under 470 nm light. I feel that the results for at least S. microadriaticum are interesting as they show a positive effect (or non-negative) effect for the blue excitation. The action spectrum for S. microadriaticum is pretty clear in that green fluorescence is rather unlikely to cause this effect but looking at the larval fluorescence spectra under similar excitation, it seems plausible to me that the orangered fluorescence might have an effect. I think this should be at least discussed although I would prefer to see an experiment on the action spectra of at least S. microadriaticum and D. trenchii with one or two filters covering those longer wavelengths and maybe one of the previous ones as a reference. I also still would like to see one more wavelength around 440-450 nm. In general, this experiment still needs some more explanation in the text to link it better to the previous experiments and an infection experiment under UV-A light should be at least mentioned in the discussion. Furthermore, I would strongly recommend linking the infection data to the ones (in white light I think?) from Yamashita et al., 2014, especially since no dark or white light controls were performed in the current manuscript.

**Our response:**

During the first round of reviews, we removed the result for the infection test under blue LED light to avoid confusion; however, in the revised manuscript, we have reincluded this result in the Appendix. Unfortunately, based on our results, it is not clear whether the orange fluorescence attracted native symbionts. Thus, we have included the discussion for orange fluorescence, and some notes about future research in the revised manuscript.

Line 513-521

*S*. *microadriaticum* cells were not acquired by *A*. *tenuis* larvae under red LED light. On the contrary, *A*. *tenuis* larvae can acquire this native symbiont under normal white light [14] or blue LED light (CWL; 470 nm, FWHM; 30 nm) (S4 Appendix). In *Acropora* *millepora* juveniles, Quigley et al. [35] found that *S*. *microadriaticum* symbionts occurred at a significantly higher abundance in redder juveniles compared to greener juveniles. The visibly greener *A*. *tenuis* larvae under fluorescent microscopy (Ex. 400–440 nm, Em. ≥475 nm; blue-violet excitation) have less orange fluorescence (S3 Appendix). Given these results, *S*. *microadriaticum* cells may be attracted to the orange fluorescence of larvae or juveniles. Nonetheless, the current phototaxis analysis was conducted using limited wavelengths because we focused on whether native symbionts possess positive phototaxis to green light. Thus, further analysis is required to conclude on the above-proposed mechanisms.

Line 579-581

To elucidate the initial attraction mechanisms, comprehensive research that includes not only green fluorescence but also other fluorescence and chemical attractants is needed.

2-5. In the supplemental datasets S2 and S3, I would highly recommend trimming the data to include only the wavelengths within the filter specs (e.g., starting only at 475 nm for blueviolet excitation and 420 nm for UV-A excitation) or, probably better, to the range shown in the graphs in Fig. 4 and Fig. 6. The authors should also consider setting negative values to zero as there cannot be less light than none.

**Our response:**

The PLOS journal requires authors to make all data related to the findings described in their manuscript fully available without restriction. Our supplemental datasets include raw data. Thus, we would like to avoid trimming or adjusting the raw data. Instead, we have included information for PMA and fluorescence filter cubes in the revised Results section.

Line 317-322

Although the PMA measured wavelength from 196 to 958 nm, data from 400 to 800 nm are plotted in Fig. 4. Furthermore, fluorescence at wavelengths shorter than 455 nm is almost undetectable due to the dichroic mirror in the U-MWBV2 filter cube. The raw data with and without correction by Fluor-Ref are shown in the supporting information (S2 dataset).

Line 339-342

Larval fluorescence spectra under UV-A excitation is shown in Fig 6 (data from 400–800 nm are plotted). Fluorescence at wavelengths shorter than 400 nm is almost undetectable due to the dichroic mirror in the U-MWU filter cube. Raw data with and without correction are shown in supporting information (S3 dataset).

2-6. I appreciate the effort of the authors to include the greyscale images as S2 and S4 Figs., but I should have been more clear; I thought of the supplemental figures to show the different color channels (red, green and for S4 Fig also blue) as greyscale separately, side-byside. After going back to the Materials & Methods and looking up the Olympus DP-73 camera and the filter cubes, I realized that this is an RGB camera and the emission filters were longpass filters. This does not affect the above comment, but I would strongly suggest including this information in the respective Material & Methods section.

**Our response:**

We apologize for the misunderstanding. In the revised manuscript, we have included a sentence describing that DP-73 is an RGB camera (Line 186 in the revised manuscript). We have also added new Appendix files (S1 appendix) for greyscale images that separate into each R, G, and B channels.

2-7. L296-298: “Although transmittance between 455 nm and 478 nm was less than 50% owing to characteristics of the emission filter, a shoulder was found between 467 nm and 507 nm (Fig 4).”

- Do the authors have access to the exact transmission characteristics of the filter so that it would be possible to correct the data for filter transmittance before plotting?

L313-314: “Owing to the characteristics of the emission filter, transmittance between 313 nm and 420 nm was less than 50%.”

- Same as previous comment.

**Our response:**

The transmittance wavelength of each optical filter within the fluorescence filter cubes can be found on OLYMPUS’ website: <https://www.olympus-lifescience.com/en/optics/mirror-units/>. We have included this information in the revised manuscript Line 323-324, and Line 344.

2-8. L381: “Although in the present study a shorter wavelength was not observed…”

- This sentence needs a bit more explanation. I think the authors mean that no phototaxis was observed at lower wavelengths for the strains used, but this is not directly clear from reading this sentence and I feel that it would also help the reader to very briefly, with half a sentence, describe the findings from the reference given here.

**Our response:**

We have modified this sentence slightly.

Line 421-423

Although a shorter wavelength was not observed in the present study, this tendency was almost congruent with the case of some dinoflagellates that showed highest phototaxis peak at blue light region (e.g., [29]).

2-9. L480-481: “Thus, various spatial scales might exist for the distance between apo-symbiotic corals and their native symbionts according to the coral life stage.”

- I don’t understand this sentence.

**Our response:**

Laboratory experiments (ours and those of other authors) have demonstrated that some Symbiodiniaceae cells can be attracted to corals in laboratory conditions; however, in natural environments, the distance between environmental algal cells and corals may be more than that in laboratory conditions using experimental bottles or cups. It is still unclear whether the attractants are effective at longer distances, such as in natural environments. We have modified this paragraph.

Line 523-535

In *Acropora* corals, after mass-spawning and subsequent fertilizations, the larvae initially swim in the water column and then settle in shaded spots on the substratum. Thus, various factors, including spacious distances and the effect of water flow, should be taken into account between or around apo-symbiotic corals and their native symbionts in the environments. A chemotaxis experiment using soft coral *H*. *fuscescens* polyps and their symbionts demonstrated that the symbiont cells swam toward the polyp against flow velocities of up to 0.5 mm s^−1^; however, the cells must be within a distance in the order of a centimeter to decimeter from the polyp to sense the chemoattractant due to the dispersion of the substance [49]. In fluorescent attraction, Aihara et al. [20] demonstrated that 8-mm^2^ *E*. *aspera* coral fragments can attract symbiodiniacean cells within a 95-mm diameter plastic dish. However, it is still unclear how far *A*. *tenuis* larvae/juveniles can attract native symbiont species using chemical and/or fluorescent attractants in the natural environments.

2-10. L516-517: “If they are within shaded locations on substrates where larvae settle, these attractions might be possible in the field.”

- Is it not much more likely that larvae acquire their symbionts in the water column while they are swimming and where the light intensity is high enough to reasonably excite the fluorescent proteins before settlement?

**Our response:**

Although we believe it is possible that the larvae could encounter symbiodiniacean cells in the water column, results from field observations are still lacking. In the water column, the distance between environmental symbiont cells and corals may be much more than that in laboratory conditions. Furthermore, the native symbiont *S*. *microadriaticum* showed phototaxis toward blue light; however, blue light is also included in the direct sunlight transmitted into the water column. Additionally, considering the water flow, it may be difficult for these algae to reach the larvae. Moreover, the number of swimming larvae peak from the fifth to the seventh day after spawning and decrease rapidly on the ninth day (namely, the larvae settles on the substratum; Suzuki et al., 2011; https://doi.org/doi: 10.3354/meps08896). This result indicates that *Acropora* larvae do not swim in the water column for very long. The chances of an encounter between symbiont cells and larvae may be limited in the water column. Future studies need to clarify how native symbiont cells encounter apo-symbiotic corals in natural environments under such situations.

2-11. Appendix S1 Fig. 2:

- From the figure legend, it is not fully clear to me, which exact peptide sequences are meant by the numbers for 1,3 as well as 4,5 where the sequences slightly differ within the RFP or CFP~GFP groups in the indicated regions.

- Additionally, I would suggest indicating the amino acid triplet which gives rise to the chromophore and maybe adding a secondary structure prediction (maybe with a wellstudied FP, e.g. AvGFP, as reference).

**Our response:**

In the revised version, each peptide was identified by a different color. Furthermore, we have indicated the chromophore with an underline. Although the secondary structure of fluorescent proteins is very important, this study does not focus on the properties of these proteins. Thus, we decided not to add secondary structures into the revised Appendix.

All of the below-mentioned suggestions have been incorporated into the revised manuscript as applicable.

1. For some references, DOIs are given and for some not. I would suggest being consistent (and in accordance with the PLoS ONE requirements).

L39-41: “…mechanisms may exist to facilitate encounter with suitable symbiont cells, by new coral generations. Green fluorescence in corals has been proposed as a mechanism to attract these cells.”

- I would suggest simplifying these sentences and to introduce phototaxis in the second sentence e.g.: “…mechanisms may exist by which new coral generations attract suitable symbionts. Phototaxis of suitable symbionts towards green fluorescence in corals has been proposed as such a mechanism.”

L66: “However, early life stages of the majority of corals that use spawn-gamete reproduction…”

- Might this be a direct word-by-word translation from Japanese? I think the usual English expression would be “broadcast spawners” (corals that release their gametes into the water column where they fertilize and then develop in contrast to brooding spawners where only sperm is released, and eggs are fertilized and develop within the parent colony and often inherit the symbionts vertically). If that assumption is correct, the sentence could be changed to e.g.: “However, early life stages of the majority of broadcast-spawning corals…”

L102: “…several Symbiodiniaceae cultured strains…” - “…several cultured Symbiodiniaceae strains…”

L129-130: “During the early to mid-logarithmic proliferation phase of each strains…” - “During the early to mid-logarithmic proliferation phase of each strain…”

L130: “(Φ 25 mm)”

- This is the Greek letter phi, there is a specific symbol for the diameter in the Unicode tables (U+2300; ⌀, unfortunately not available in most common fonts; but U+00F8, ø, should be a valid substitute in most fonts).

L177: “epifluorescent microscope” - Must be “epifluorescence microscope”.

L178-179: “Fluorescence filter cubes were U-MWBV2 (Ex. 400–440 nm, Em. 475 nm; blueviolet excitation) and U-MWU (Ex. 330–385 nm, Em. 420 nm; UV-A excitation).”

- I would suggest clarifying that the emission filters were longpass filters in contrast to the excitation bandpass filters by i.e.: “Fluorescence filter cubes were U-MWBV2 (Ex. 400–440 nm bandpass, Em. ≥ 475 nm longpass; blue-violet excitation) and U-MWU (Ex. 330–385 nm bandpass, Em. ≥ 420 nm longpass; UV-A excitation).” In addition, I would suggest adding the information that the camera is an RGB-color camera to line 180-181 (see also minor general comment 3. on Figs. S2 and S4).

L183-185: “…set at on the camera port of the microscope.”

- Maybe change to: “…attached to the camera port of the microscope.”

L194: “…cause saturation of PMA under with settings used.”

- “…cause saturation of the PMA with the used settings.”

L211: “…red LED…”

- This occurs a number of times, I would recommend writing “red LED light”.

L225: “…epifluorescent microscope (BX50; 400−410 nm excitation)…”

- This must be “epifluorescence microscope”. Also please add the emission filter properties.

L284: “Fig 2. The phototaxis patterns s of Symbiodiniaceae cultured strains.”

- I would suggest “Fig 2. The phototaxis patterns of cultured Symbiodiniaceae strains.”

L284-286: “The 100% line indicates that initial cell density and cell density after light irradiation if not changed.”

- This sentence needs rephrasing. I would suggest something like “The 100% line indicates constant cell densities before and after irradiation.”

L315: “Fluorescence intensities under UV-A were overall lower than under blue-violet.”- “Fluorescence intensities under UV-A excitation were overall lower than under blueviolet excitation.”

L321-322: “…are found in A. tenuis genome (S1 Appendix). These six-fluorescent…”

- “…are found in the A. tenuis genome (S1 Appendix). These six fluorescent…”

L329: “…Em. 375 nm).” - “…Em. ≥375 nm).”

L333: “…Em. 375 nm).” - “…Em. ≥375 nm).”

L339: “…Em. 420 nm).” - “…Em. ≥420 nm).”

L344: “…Em. 420 nm).” - “…Em. ≥420 nm).”

L358: “…significantly differed depended…”

- “…significantly differed dependent…”

L376-377: “Native symbiont species S. microadriaticum cultured strain…”

- I would suggest a change to e.g., “Cultured algae of the native symbiont S. microadriaticum…”

L378-379: “A similar trend was also found in free-living species S. natans cultured strain…”

- Change to e.g. “A similar trend was also found in cultures of the free-living species S. natans…”

L409: “A simple compare” - “A simple comparison”

L411: “…with that of reported by…” - “…with that reported by…”

L449-450: “The light environment in these shaded spots is thought mainly scattered and reflected light rather than direct sunlight.”

- “The light environment in these shaded spots is thought to consist of mainly scattered and reflected light rather than direct sunlight.”

L458: “...infection test was conducted under red LED light.”

- “...infection tests were conducted under red LED light.”

L469: “…cells of S. microadriaticum cultured strain…”

- “…cells from S. microadriaticum cultures…”

L474: “Although a longer wavelength was not observed…”

- Just a bit more precise: “Although phototaxis response to a longer wavelength than 570 nm was not assessed…”

L483: “…however, it is unclear these attractants are effective at longer distance.”

- “…however, it is unclear if these attractants are effective at longer distance.”

L494: “The peak wavelength…” - “At the peak wavelength…”

L521: “…are needed.” - “…is needed.”

Filename: “new_S2_dataset_fluorescence spectra under biolet-bule.xlsx”

- “new_S2_dataset_fluorescence spectra under violet-blue.xlsx”

- And just a minor thing: I believe it must be “conserved” and not “conservative” in the last line of the legend.
