# Supplementary material for: Can Acropora tenuis larvae attract native Symbiodiniaceae cells by green fluorescence at the initial establishment of symbiosis?
Source: PLoS One. 2021 Jun 1;16(6):e0252514. doi: 10.1371/journal.pone.0252514 (PMC8168901; doi:10.1371/journal.pone.0252514)
Supplement: Attachment — Submitted filename: Response_to_Reviewer_3rd.docx [file pone.0252514.s016.docx]

The manuscript has been revised in accordance with the comments provided by the reviewer. This letter contains the details of our revisions. Additionally, we have replaced one reference, because the original reference no.48 is paper of Symposium Proceedings, but the full paper is now accepted. This replace do not affect the discussion.

Original reference no.48

Takeuchi R, Jimbo M, Tanimoto F, Kuniya N, Yamashita H, Suzuki G, Yasumoto K, Watabe S (2017) Juvenile *Acropora tenuis* attract *Symbiodinium* by using GlcNAc-binding lectin. The JSFS 85th Anniversary-Commemorative International Symposium. Symposium Proceedings, No. 12003.

New reference no.48

Takeuchi R, Jimbo M, Tanimoto F, Iijima M, Yamashita H, Suzuki G, Harii S, Nakano Y, Yasumoto K, Watabe S (Accepted) N-Acetyl-D-Glucosamine-binding lectin in *Acropora* *tenuis* attracts specific Symbiodiniaceae cell culture strains. Mar Drugs.

**Major general comments:­**

1. L277-295: Looking at the graphs in Fig. 2, I think the authors need to discuss here not only the statistical significance but also the possible biological one. Especially the differences for *B. minutum* are so minimal that I would be really surprised if they had any relevance for attraction (even though they are statistically significant). The opposite seems true for *D. trenchii*, where the variance between replicates prohibits any statistical significance but there might be an actual underlying pattern. In my opinion, this should be mentioned in one or two additional sentences.

**Our response:**

Thank you for your suggestion. We have included several sentences related to the reviewer’s opinion in the revised “Results” section.

Lines 290–299

In *Breviolum* sp., weak double peaks for the positive phototaxis action spectrum (358 nm and 461 nm) were predicted. The relationship between the wavelength and phototaxis was statistically significant in the cultured strains of *S*. *tridacnidorum*, *Breviolum* sp., and *F*. *kawagutii*, however, we could not observe strong positive phototaxis for a specific wavelength in these species (Fig. 2). Conversely, in another native symbiont *D*. *trenchii*, although the cells appeared to have a positive phototaxis to 470 nm light (Fig. 2), we could not describe a statistically supported relationship (edf = 2.074, F = 1.261, adj. R^2^ = 0.115, *p* = 0.266). We could not find significant relationship between the wavelength and phototaxis in the case of the *C*. *goreaui* and *E*. *voratum* cultured strains either (edf = 2.006 and 3.048, F = 2.485 and 0.389, adj. R^2^ = 0.175 and 0.0251, *p* = 0.125 and 0.681, respectively).

**Specific comment 1.**

L58-61: “Thus, attraction mechanisms other than visible fluorescence may exist, at least for *D*. *trenchii* . Our results suggest that attraction mechanisms other than green fluorescence could exist, a topic which requires further investigation and discussion.”

- The two sentences are mostly redundant. I would recommend collapsing them into one sentence.

**Our response:**

We have slightly modified this sentence.

Lines 58–61

Thus, attraction mechanisms other than visible fluorescence might exist, at least in the case of *D*. *trenchii*. Our results suggest that further investigation and discussion, not limited to green fluorescence, would be required to elucidate the initial attraction mechanisms.

**Specific comment 2.**

L305-309: - I think, this information is important but is sufficient to include only in the figure legends. Therefore, I would recommend to completely delete this paragraph.

**Our response:**

We have removed this part.

**Specific comment 3.**

L351-354:- I highly recommend moving this part on the proteomics to the beginning of the section (before L310).

**Our response:**

We agree with this comment. We have slightly modified and moved this part to the beginning of this section.

Lines 309–312

In the *A*. *tenuis* genome, at least three cyan-green and three red fluorescent protein-like protein-encoding genes are found (S1 Appendix). These six-fluorescent protein-like proteins are also detected in apo-symbiotic *A*. *tenuis* larvae as peptides during proteomic analysis (S1 Appendix).

**Specific comment 4.**

L574: “These results may hint toward a specific attraction mechanism.”

- Did the authors mean a “specific chemical attraction mechanism”?

**Our response:**

We apologize for the lack of explanation. We have slightly modified this sentence.

Lines 568–574

For example, fluorescence variation could be associated with preferential uptake of Symbiodiniaceae types [35]. In the case of soft coral, motile symbiont cells are attracted to solutions containing exudates of apo-symbiotic juvenile but not adult polyps [19]. These results could potentially provide important information to clarify the mechanisms underlying the specific attraction of certain Symbiodiniaceae types or species toward larvae/juvenile corals.

**All of the below-mentioned suggestions were incorporated into the revised manuscript as applicable.**

L48: “Although the larvae fluoresced with green and broad orange fluorescence…”

- I would suggest changing this to “Although the larvae exhibited green and broad orange fluorescence…”

L81: “…some mechanism likely exists…”

- I suggest changing this to “…some mechanisms likely exist…” to account for the possibility of multiple parallel mechanisms for attraction.

L82: “Laboratory experiments show that some Symbiodiniaceae cultured strains…”

- Should be “Laboratory experiments show that some cultured Symbiodiniaceae strains…”

L87-88: “…juvenile polyps of soft coral Heteroxenia fscescens.”

- Better: “…juvenile polyps of the soft coral Heteroxenia fuscescens.”

L88-89: “…green fluorescence protein (GFP)…”

- “…green fluorescent protein (GFP)…”

L222: “…following Suzuki [25].”

- I think, this must be “…following Suzuki, 2020 [25].”

L240: “…we used generalized additive model…”

- This should be “…we used the generalized additive model…”

L313: “…orange fluorescence was observed after 2-day-old larvae.”

- Better: “…orange fluorescence was observed after 2 days.”

L338-339: “…even in the 1-day-old coral. The orange fluorescence was also observed more than the 3-day-old larvae.”

- I would suggest writing: “…even in the 1-day-old larvae. Orange fluorescence was observed in larvae older than 3 days.”

L349: “…however, a 522 nm green fluorescence peak…”

- “…however, the 522 nm green fluorescence peak…”

L359: “Micrographs 1 to 3-day-old larvae…”

- “Micrographs of 1 to 3-day-old larvae…”

L425-426: “…, and calculations based on the thickness of each layer, it is ideal for reflection of light with wavelengths of 320–520 nm [23].”

- I would recommend changing this to: “…, and calculations based on the thickness of each layer indicate that it is ideal for reflection of light with wavelengths of 320–520 nm [23].”

L440: “…E. aspera coral emits…”

- “…E. aspera corals emit…”

L441: “cultured symbiodiniacean strain “OTcH-1” shows”

- Remove the quotation marks.

L458: “A broad “orange” fluorescence”

- Remove the quotation marks.

L502-504: “However, genus Durusdinium including D. trenchii were often found within the Acropora larvae/juveniles [10,13,14,46,47].”

- I would suggest “However, genus Durusdinium including D. trenchii is often found　within Acropora larvae/juveniles [10,13,14,46,47].”

L527-528: “A chemotaxis experiment using soft coral H. fuscescens polyps…”

- Better: “A chemotaxis experiment using polyps of the soft coral H. fuscescens…”

S2 Appendix Fig. 2 description: “The black bar indicate chromophore.”

- Should be: “The black bar indicates the chromophore.”

S3 Appendix: “…observed under an epifluorescent microscope (BX50, Olympus, Tokyo,

Japan).”

- Should be: “…observed under an epifluorescence microscope (BX50, Olympus,

Tokyo, Japan).”
