# Supplementary material for: Can Acropora tenuis larvae attract native Symbiodiniaceae cells by green fluorescence at the initial establishment of symbiosis?
Source: PLoS One. 2021 Jun 1;16(6):e0252514. doi: 10.1371/journal.pone.0252514 (PMC8168901; doi:10.1371/journal.pone.0252514)
Supplement: Attachment — Submitted filename: Response_to_Academic editor_4th.docx [file pone.0252514.s017.docx]

The manuscript has been revised in accordance with the comments provided by the academic editor. This letter contains the details of our revisions. Additionally, we have replaced one reference, because a recent paper identified a new clade, “clade J”. The family Symbiodiniaceae now includes ten clades, A–J. This replacement does not affect the discussion.

**Original reference no.3**

Pochon X, Gates RD (2010) A new Symbiodinium clade (Dinophyceae) from soritid foraminifera in Hawai’i. Mol Phylogenet Evol 56: 492–497.

**New reference no.3**

Yorifuji M, Yamashita H, Suzuki G, Kawasaki T, Tsukamoto T, Okada W, Tamura K, Nakamura R, Inoue M, Yamazaki M, Harii S (2021) Unique environmental Symbiodiniaceae diversity at an isolated island in the northwestern Pacific. Mol Phylogenet Evol. https://doi.org/10.1016/j.ympev.2021.107158.

**Comments from Academic editor:­**

1. Please mention the nature of the error bars in the figures: standard deviation?

**Our response:**

We have included the nature of the error bars in the Figure legend of Fig.2. “The mean and ~~SD~~ standard deviation of triplicate measurements (error bar) are shown.”

6. When providing exact p-values, use the "=" instead of ">" or "<" (which should be reserved for when you want to emphasize whether the value is above or under some preset threshold (e.g. 0.05).

**Our response:**

We used "<" for one of the p-values in the Results section, because the result of the calculation was extremely small and was below the descriptive limit of the R system and the package used. If we use “=” instead of “<” for this value, the calculation results would not be accurate. Thus, we would like to leave the reporting as it stands.

8. Lines 244-245. Why use a mixed-effect model? Is this because you tracked individuals, in which case there were repeated measures? I would state the justification for this in the text.

**Our response:**

We sacrificed and observed three larvae each for each observation. The larvae were kept in the incubator at a constant temperature and light intensity. However, different larvae develop at slightly different speeds. Furthermore, the experimental larvae were originally from nine parental coral colonies; therefore, a variety of parental combinations was possible. These differences in the larvae may affect the fluorescence intensity in ways that are difficult to quantify. Thus, we included a random effect to take account of variations in larval fluorescence intensity among the individual larvae. Accordingly, we have slightly modified the sentence in the statistical analysis section.

Lines 255–260

The intensity was assumed to follow a gamma distribution, and the link function was log; the explanatory variable was larval age; ~~the random effect was assumed to vary among the individual larvae;~~ the fluorescence intensity of the fluorescence reference slides (GREEN) at 522.4 nm was used as an offset. The random effect was assumed to vary among individual larvae; since, the fluorescence intensity of individual larvae may be affected by the individual-specific characteristics, such as development speed and/or parental combinations.

Lines 272–274

Since the infection rates may be affected by the environments in the individual experimental glass cup, the random effect was assumed to vary among the experimental glass cups.

9. Lines 211-212: Do you mean that larvae were alive (vs. fixed in formalin)? If so, I would rephrase this for clarity.

**Our response:**

In the present study, the observations were conducted on live larvae without fixation. We have added a sentence to clarify this issue.

Lines 215–217

All observations were conducted without fixations, i.e., fluorescent micrographs and fluorescence spectra were obtained from live larvae.

15. For Figure 1d (super cool!), would it be possible to put a scale bar on the right of panel? It is hard to know is that part of the image is at the same scale as the flask. It's actually hard to tell what is being shown in general, but I think the figure caption explains it.

**Our response:**

Thank you for your comment. Fig. 1d shows how each wavelength of light passes though the cell culture flask. This photograph was taken from the upper diagonal, and the image has depth. Thus, it is difficult to add a scale to this figure. Instead, we have included a scale bar in Fig 1a. In addition, we have included the size of each optical filter (ø 25 mm) in the figure legend of Fig 1b. These changes would make the size of the cell culture flask clearer.

17. Consider putting the statistical findings (e.g., ANOVA) in a consolidated table (Table 1).

**Our response:**

In the statistical analysis section, we have described “using the anova function in R.” However, this command in R does not calculate the analysis of variance (ANOVA). We used this command to compare the null and the full models, i.e., it is a likelihood ratio test. The statistical values from the likelihood ratio tests are included with the appropriate p-values in the Results section. For example “The intensity of this green fluorescence significantly differed by larval age (Δdeviance = 40.58, Δdf = 1, *p* = 1.885 × 10^−10^). The percentage of Symbiodiniaceae cell infected larvae significantly differed depending on the supplied species (Δdeviance = 5.6493, Δdf = 1, *p* = 0.01746).”

**All of the below-mentioned edits were incorporated into the revised manuscript.**

2. When using numbers <10, you should write them out, i.e., "two" instead of "2."

3. "aposymbiotic" is not hyphenated.

4. Since corals have many different symbionts (bacterial, dinoflagellate, and other), and you are ONLY discussing the Symbiodiniaceae, you should refer to them as "endosymbionts" instead of "symbionts." Similarly, avoid using "algal" but instead use "dinoflagellate" or "Symbiodiniaceae."

5. You only need to mention the full genus name the first time you use it. Afterwards, it can be abbreviated.

7. Sometimes you abbreviated figure as "Fig" and sometimes as "Fig." Check a recent issue of PLoS ONE to see the preferred nomenclature and maintain consistency throughout.

10. Line 199: No need to capitalize words in websites.

11. Lines 187-188: Since you mention a complete sentence i parentheses, you need a period (".") prior to the closing parentheses (i.e., ".)").

12. Line 183 and elsewhere: The semicolon should come directly after a word, not after a space. I think this may be because you used the justified setting (it should be the leftwards orientation instead).

13. Sometimes you use "Supplemental S1 dataset" and sometimes "S1 dataset." I think PLoS ONE prefers the latter.

14. Lines 122-123: This statement needs a reference.

16. Figure 7: awesome! I have never heard of a sunflower plot in my life, but I like it.

18. Lines 360-362: This should be written in past tense.

19. Line 341: Two periods (only need one).

20. Line 318: 50-ms (since it is being used as an adjective).
